# Targeting fibroblast CD248 attenuates CCL17-expressing macrophages and tissue fibrosis

**DOI:** 10.1038/s41598-020-73194-x

**Published:** 2020-10-08

**Authors:** Chen-Hsueh Pai, Shu-Rung Lin, Chia-Hao Liu, Szu-Yu Pan, Hao Hsu, Yi-Ting Chen, Ching-Tzu Yen, I-Shing Yu, Hua-Lin Wu, Shuei-Liong Lin, Shu-Wha Lin

**Affiliations:** 1 Department of Clinical Laboratory Sciences and Medical Biotechnology, College of Medicine, National Taiwan University, Taipei, 100 Taiwan; 2 Department of Bioscience Technology, College of Science, Chung-Yuan Christian University, Taoyuan, Taiwan; 3 Center for Nanotechnology and Center for Biomedical Technology, Chung-Yuan Christian University, Taoyuan, Taiwan; 4 Graduate Institute of Physiology, College of Medicine, National Taiwan University, Taipei, 100 Taiwan; 5 Department of Internal Medicine, Far-Eastern Memorial Hospital, New Taipei City, Taiwan; 6 Department of Internal Medicine, College of Medicine, National Taiwan University Hospital, National Taiwan University, Taipei, Taiwan; 7 Department of Integrated Diagnostics and Therapeutics, National Taiwan University Hospital, Taipei, Taiwan; 8 Laboratory Animal Center, College of Medicine, National Taiwan University, Taipei, Taiwan; 9 The Institute of Basic Medical Sciences, College of Medicine, National Cheng Kung University, Tainan, Taiwan; 10 Research Center for Developmental Biology and Regenerative Medicine, National Taiwan University, Taipei, Taiwan

**Keywords:** Renal fibrosis, Mechanisms of disease

## Abstract

The role of fibroblasts in tissue fibrosis has been extensively studied. Activated fibroblasts, namely myofibroblasts, produce pathological extracellular matrix. CD248, a type I transmembrane glycoprotein, is expressed in fibroblasts after birth. In human chronic kidney disease, upregulated CD248 in myofibroblasts is linked to poor renal survival. In this study, we demonstrated a novel interaction between CD248 and macrophages to be a key step in mediating tissue fibrosis. CD248 was upregulated in myofibroblasts in murine models of renal and peritoneal fibrosis. *Cd248* knockout (*Cd248*^*–/–*^) could attenuate both renal and peritoneal fibrosis. By parabiosis of GFP reporter mice and *Cd248*^*–/–*^ mice, we showed that attenuation of renal fibrosis was associated with a decrease of macrophage infiltration in *Cd248*^*–/–*^ mice. Moreover, decrease of *chemokine* (*C–C motif*) *ligand 17* and *Ccl22* was found in macrophages isolated from the fibrotic kidneys of *Cd248*^*–/–*^ mice. Because galectin-3-deficient macrophages showed decreased *Ccl17* and *Ccl22* in fibrotic kidneys, we further demonstrated that CD248 interacted specifically with galectin-3 of macrophages who then expressed CCL17 to activate collagen production in myofibroblasts. Mice with DNA vaccination targeting CD248 showed decreased fibrosis. We thus propose that CD248 targeting should be studied in the clinical tissue fibrosis setting.

## Introduction

CD248, also known as tumor endothelial marker 1 or endosialin, is a type I transmembrane glycoprotein that is expressed in stromal cells in normal tissues and cancers^1,2^. Using *lacZ* knock-in (+ */lacZ*) mice, we have shown that *Cd248* expression decreases in most organs, but increases and persists in the kidneys postnatally, specifically in glomerular mesangial cells and perivascular cells^2^. CD248 has also been identified in normal kidney pericytes and perivascular fibroblasts of mice by specific antibody staining^3^. In human chronic kidney disease, upregulated CD248 in myofibroblasts is linked to poor renal survival^3^. Many studies, including ours, have identified pericytes and perivascular fibroblasts as the major progenitor cells of scar-producing myofibroblasts during renal fibrosis^4–12^. The mechanisms underlying the impact of increased CD248 expression in myofibroblasts, namely activated pericytes or fibroblasts, on kidney disease progression is not clear.

In addition to the expanded population of myofibroblasts in damaged tissue, inflammatory monocytes are selectively recruited and differentiate into distinctive subpopulations of macrophages^13–18^. An increasing amount of evidence indicates that in the case of long-term, recurrent damage to tissues, sustained macrophage infiltration often results in the continuous production of various wound-healing growth factors that ultimately cause additional pathology, including parenchymal tissue destruction, microvascular rarefaction and irreversible fibrosis^13–16,18^. Recruited, not resident, macrophages have been shown to play a major role in the progression of organ fibrosis^14,16,18,19^. In addition to being the master regulators of immune cells involved in tissue destruction and remodeling in sterile inflammatory organs, macrophages produce a broad range of paracrine-signaling cytokines, thereby stimulating neighboring scar-producing myofibroblasts to proliferate and synthesize pathological extracellular matrix proteins, such as collagen I^4,14,17–20^. In murine progressive fibrosis models, including those for the kidney, liver, lung, skin and peritoneum, the examination of monocyte trafficking has shown that circulating Ly6C^high^ pro-inflammatory monocytes are selectively recruited to injured tissues and differentiate into an Ly6C^low^ pro-fibrotic population^14,18,21–24^. Using a genetic model of macrophage ablation in mice, we have demonstrated the general roles of macrophages, especially the pro-fibrotic role of recruited macrophages when they differentiate into a Ly6C^low^ population, in renal and peritoneal fibrosis^14,18^. Subpopulations of macrophages have been classified according to cell-surface protein expression^13,14,17,19,25^. Macrophages can be assigned to a pro-inflammatory phenotype (M1 or classical activation) or a pro-fibrotic phenotype (M2 or alternative activation) according to their distinctive cytokine profiles and behavior following activation^13,14,19,25^. Although specific stimulation by lipopolysaccharide (LPS) and interferon γ (IFNγ) or interleukin (IL)-4/IL-13 can induce distinctive macrophage subpopulations in vitro, the mechanisms underlying the phenotype switch of recruited macrophages in vivo remain obscure^14,17,26^.

Evidence has shown that macrophages produce paracrine-signaling cytokines to activate myofibroblasts for scar production^4,14,17–20^, but whether myofibroblasts can induce phenotype switch of macrophages remains obscure^14,17,26^. We here demonstrated the role of CD248 in activation of myofibroblasts and pro-fibrotic phenotype switch of macrophages during progressive fibrosis.

## Results

### Targeted disruption of the *Cd248* gene attenuates tissue fibrosis

**Figure 1:**
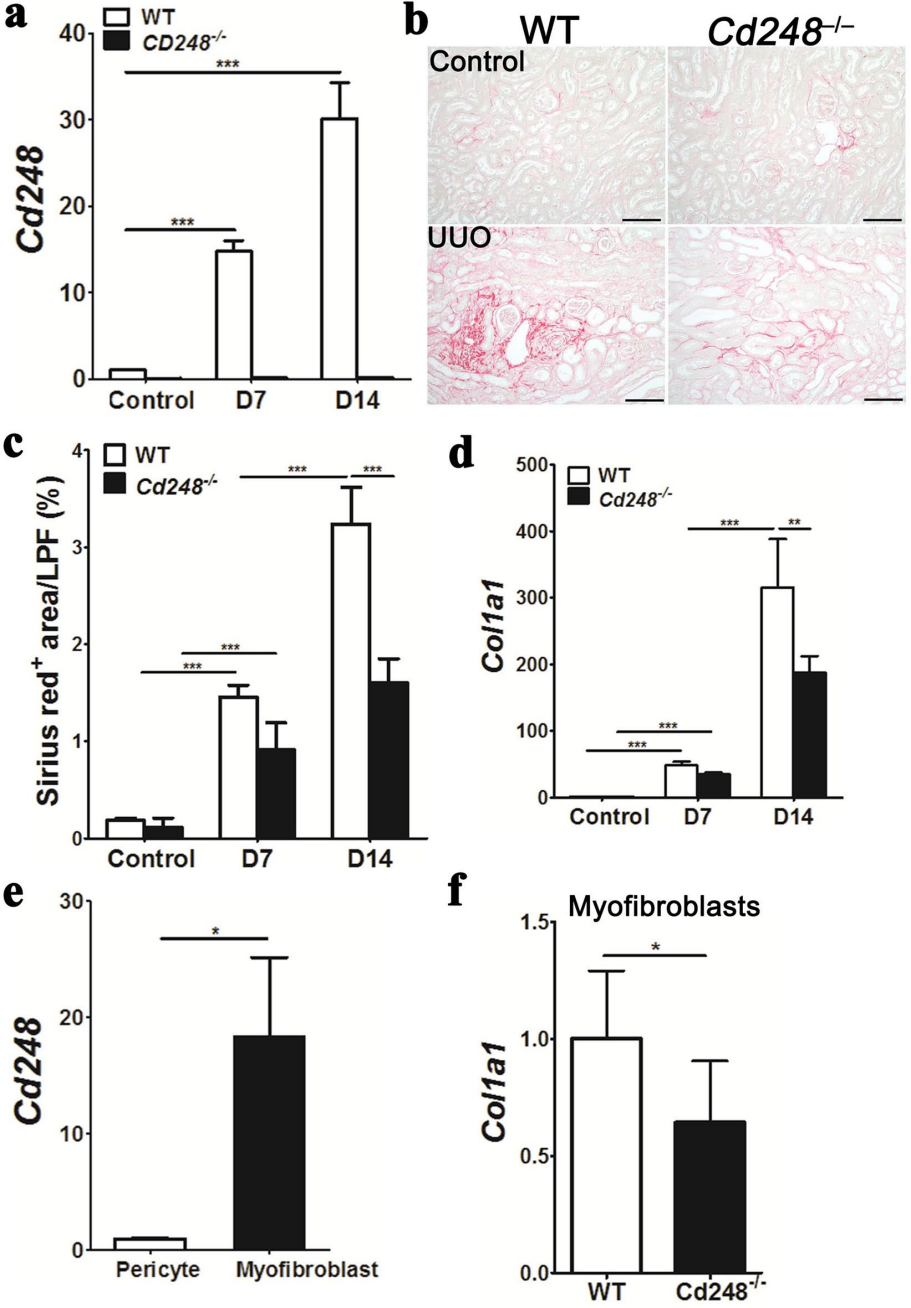
*Cd248* disruption attenuated murine obstructive renal fibrosis. (**a**) Quantitative polymerase chain reaction (qPCR) of renal *Cd248* in mice of wild type (WT) and CD248 knockout (*Cd248*^*–/–*^) before (control), on day 7 (D7) and day 14 (D14) after unilateral ureteral obstruction (UUO) surgery. *Cd248* expression was normalized by *glyceraldehyde 3-phosphate dehydrogenase* (*Gapdh*) and then compared with that of WT control. *n* = 10. (**b**) Representative images of picrosirius red staining in kidneys before (control) and on D14 after UUO surgery. Original magnification, × 100. Scale bar, 100 μm. (**c**) Quantification of Sirius red^+^ collagen fibrils on low-powered field (LPF) images of kidney sections taken at 100× magnification. *n* = 10. (**d**) qPCR of renal *Col1a1*, which encoded the collagen I α1 chain. *n* = 6–10. (**e**) qPCR of *Cd248* in pericytes and myofibroblasts isolated from control and D7 UUO kidneys of *Col1a1-GFP*^*Tg*^ mice, respectively. *n* = 6. (**f**) qPCR of *Col1a1* in myofibroblasts isolated from D7 UUO kidneys of WT and *Cd248*^*–/–*^ mice. *n* = 6. Data are expressed as means ± standard errors of the mean. ***P < 0.001 by one-way ANOVA with post hoc Tukey’s multiple comparisons test in (**a**,**c**,**d**) and *P < 0.05 by unpaired t-test in (**e**,**f**).

CD248 involvement in tissue fibrosis was first examined in a renal fibrosis model induced by unilateral ureteral obstruction (UUO). *Cd248* transcript was upregulated in UUO-induced fibrotic kidneys of WT, but not in *Cd248*^*lacZ/lacZ*^ (hereafter *Cd248*^*–/–*^) mice (Fig. 1a). Loss of CD248 resulted in reduced deposition of Sirius red^+^ collagen (Fig. 1b,c, Supplementary Figure S1), and reduced upregulation of *Col1a1* transcript in UUO-induced fibrotic kidneys (Fig. 1d). In transgenic *Col1a1-GFP*^*Tg*^ mice who synthesized green fluorescence protein (GFP) under the control of collagen I α1 chain promoter, CD248 expression was confirmed in both Col1a1-GFP^+^ control kidney pericytes and UUO-induced kidney myofibroblasts (Supplementary Figure S2). By fluorescence-activated cell sorting (FACS) from *Col1a1-GFP*^*Tg*^ mice, pericytes and myofibroblasts were isolated from the control kidney and UUO fibrotic kidney, respectively. Specifically, *Cd248* transcript was detected in the pericytes, but markedly upregulated in myofibroblasts (Fig. 1e). We then crossed *Col1a1-GFP*^*Tg*^ mice with *Cd248*^*–/–*^ mice, such that the offspring would have GFP-expressing *Cd248*^*–I–*^ UUO-kidney myofibroblasts. FACS-isolated GFP^+^ UUO-kidney myofibroblasts showed that *Col1a1* was upregulated in WT mice but attenuated in *Cd248*^*–/–*^ mice (Fig. 1f). These data show that collagen expression in renal myofibroblasts was reduced by the loss of their own CD248.

In the second renal fibrosis model induced by unilateral ischemia–reperfusion injury (uIRI), *Cd248* disruption resulted in reduced collagen deposition and fibrosis, too (Supplementary Figure S3). CD248 involvement in tissue fibrosis was further examined in a murine model of chemical-induced peritoneal fibrosis by intraperitoneal injection of sodium hypochlorite to mimic severe fibrosis in encapsulating peritoneal sclerosis as previously described^27^. We observed accumulated *Cd248*-expressing fibroblasts in injured peritoneum (Supplementary Figure S4). Again, *Cd248* disruption attenuated peritoneal adhesion and fibrosis (Supplementary Figure S5a–c). Together, these results identified that CD248 plays a significant role in tissue fibrosis and *Cd248* disruption attenuates fibrosis.

### *Cd248* disruption does not affect cell numbers or proliferation of myofibroblasts

**Figure 2:**
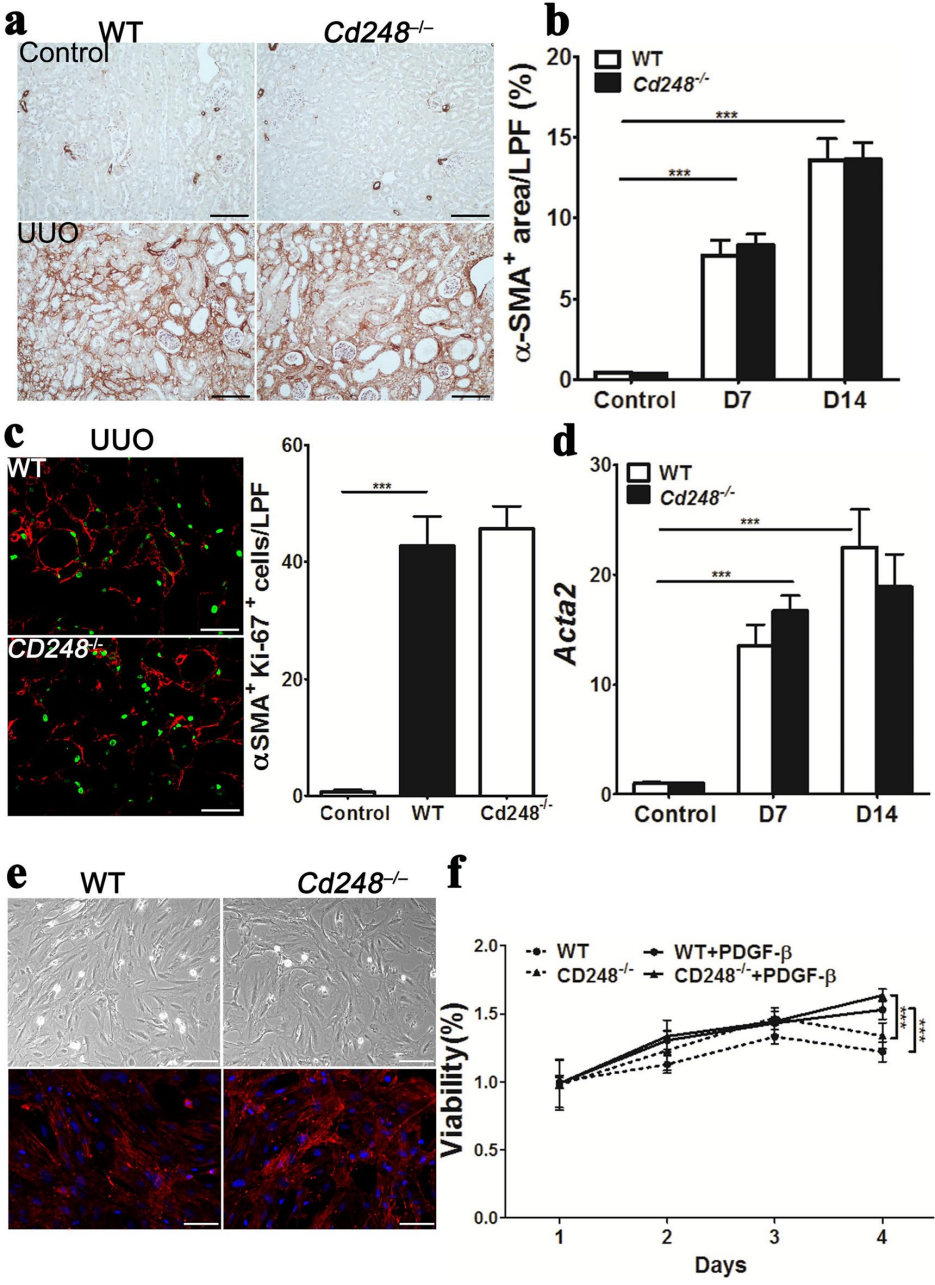
*Cd248* disruption did not affect proliferation of kidney myofibroblasts. (**a**) Representative images of α-smooth muscle actin (αSMA) immunostaining for myofibroblasts in control and D14 UUO kidneys. αSMA^+^ staining was brown. Original magnification, × 100. Scale bar, 100 μm. (**b**) Quantification of αSMA^+^ areas on LPF images of kidney sections taken at 100× magnification. *n* = 6. (**c**) Representative images of immunostaining for αSMA (red) and Ki67 (green) in D7 UUO kidneys. Original magnification, × 100. Scale bar, 100 μm. Bar chart showing the number of αSMA^+^Ki67^+^ cells/LPF. *n* = 6. (**d**) qPCR of renal *Acta2*, which encoded αSMA. *n* = 6. (**e**) Representative images for bright-field (upper panel) and αSMA (lower panel) staining of cultured myofibroblasts isolated from D7 UUO kidneys. Original magnification, × 100. Scale bar, 100 μm. (**f**) Platelet-derived growth factor-B stimulated proliferation of cultured myofibroblasts, as determined by 3-(4, 5-dimethylthiazolyl-2)-2, 5-diphenyltetrazolium bromide assay. The *y* axis shows the optic density (OD550) relative to day 0. *n* = 5. Data were expressed as means ± standard errors of the mean. ***P < 0.001 by one-way ANOVA with post hoc Tukey’s multiple comparisons test.

To investigate whether reduced fibrosis in UUO kidneys of *Cd248*^*–/–*^ mice was due to fewer scar-producing myofibroblasts, we analyzed cell numbers and their proliferation ability. Immunolabeling studies revealed no differences in α-smooth muscle actin (αSMA)^+^ area and Ki-67^+^αSMA^+^ cells between *Cd248*^*–/–*^ and WT UUO kidneys, indicating that loss of *Cd248* did not affect cell numbers and proliferation of myofibroblasts in UUO kidneys (Fig. 2a–c). Upregulation of *Acta2* transcripts, which encoded αSMA, in UUO kidneys was not affected by loss of *Cd248* (Fig. 2d). We quantified the cell numbers of αSMA^+^Col1a1-GFP^+^ myofibroblasts and αSMA^–^Col1a1-GFP^+^ pericytes in the UUO kidneys of *Col1a1-GFP*^*Tg*^ mice with or without *Cd248* knockout on day 7 after surgery (Supplementary Figure S6). No difference in the cell numbers was found, confirming again that loss of CD248 did not affect the cell proliferation of pericytes/myofibroblasts after UUO injury. *Cd248* disruption did not affect the cell numbers of αSMA^+^ myofibroblasts in fibrotic peritoneum, either (Supplementary Figure S5d, e). Platelet-derived growth factor (PDGF)-B-stimulated proliferation did not differ between *Cd248*^*–/–*^ and WT myofibroblasts isolated and cultured from UUO kidneys (Fig. 2e,f). Therefore, the reduction of fibrosis in UUO kidneys of *Cd248*^*–/–*^ mice could not be attributed to a reduction in myofibroblast numbers or their proliferation potential.

### *Cd248* affects macrophage infiltration and pro-fibrotic phenotype switching in injured organs

**Figure 3:**
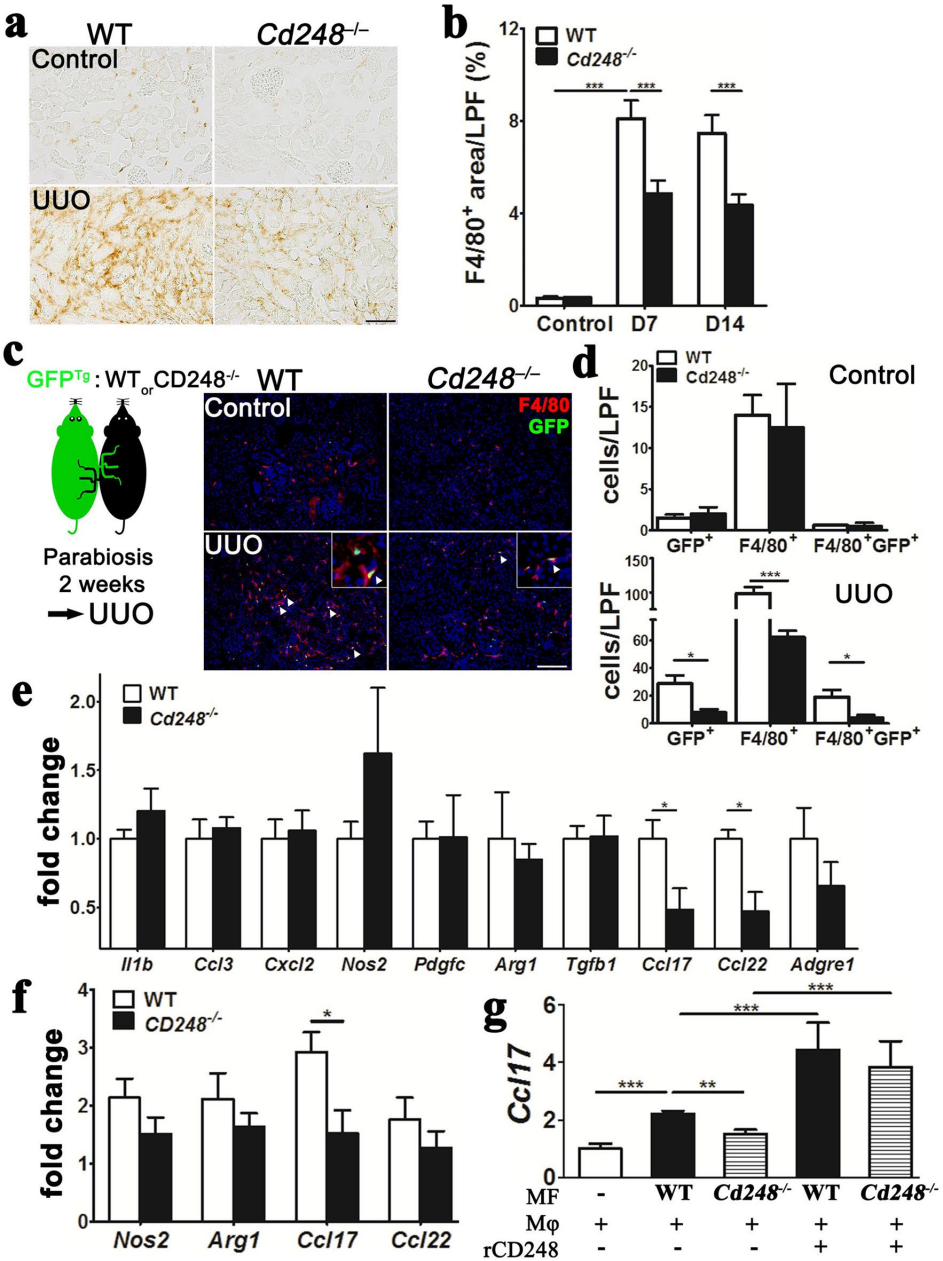
*Cd248* disruption reduced infiltration and pro-fibrotic phenotype switching of kidney macrophages during renal fibrosis. (**a**) Representative images of F4/80 immunostaining for macrophages in control and D14 UUO kidneys. F4/80^+^ staining was brown. Original magnification, × 100. Scale bar, 100 μm. (**b**) Quantification of F4/80^+^ areas on LPF images of kidney sections taken at 100 × magnification *n* = 10. (**c**). Representative images of green fluorescent protein (GFP)^+^ circulation-derived cells and F4/80^+^ (red) macrophages in control and day 3 (D3) UUO-kidneys of WT and *Cd248*^*–/–*^ parabionts joined surgically to transgenic *GFP* (*GFP*^*Tg*^) mice. Arrowhead indicates an F4/80^+^GFP^+^ macrophage. Original magnification, × 100. Scale bar, 100 μm. (**d**) Quantification of GFP^+^, F4/80^+^ and F4/80^+^GFP^+^ cells on LPF images of control (upper panel) and D3 UUO (lower panel) kidneys of WT and *Cd248*^*–/–*^ parabionts. *n* = 3. (**e**) qPCR of genes encoding cytokines and enzymes in macrophages isolated from D7 UUO kidneys. *n* = 4. (**f**) qPCR of *Nos2*, *Arg1* and *Ccl17* in lipopolysaccharide (LPS) and interferon γ (IFNγ)–primed RAW264.7 macrophages co-cultured with D7 UUO-kidney myofibroblasts isolated from WT or *Cd248*^*–/–*^ mice in the Transwell system. RAW264.7 macrophages co-cultured with medium were used as control. *n* = 4. (**g**) qPCR of *Ccl17* in WT bone marrow–derived macrophages (BMDMs, Mϕ) co-cultured with medium only (control) or with WT or *Cd248*^*–/–*^ UUO-kidney myofibroblasts (MF) in the same dish. Recombinant CD248 (rCD248) was included in the culture as indicated. *n* = 5. Data are expressed as means ± standard errors of the mean. *P < 0.05, **P < 0.01, ***P < 0.001 by one-way ANOVA with post hoc Tukey’s multiple comparisons test in (**b**,**g**) and unpaired t-test in (**d**,**e**,**f**).

We observed marked accumulation of F4/80^+^ macrophages in UUO kidneys, but macrophage accumulation was notably reduced in the UUO kidneys of *Cd248*^*–/–*^mice (Fig. 3a,b). Attenuation of macrophage accumulation in injured peritoneum was also found in *Cd248*^*–/–*^mice (Supplementary Figure S5f–h).

In parabiosis experiments designed to investigate monocyte recruitment, each transgenic *CAG-EGFP* mouse, hereafter referred to as *GFP*^*Tg*^, was surgically joined to a WT or *Cd248*^*–/–*^mouse. FACS showed that > 50% of circulating CD45^+^ cells expressed GFP in both WT and *Cd248*^*–/–*^parabionts 14 days postoperatively, demonstrating successful cross-circulation (Supplementary Figure S7). Relative to the UUO kidneys of WT parabionts following UUO surgery, immunofluorescence studies showed notably reduced cell numbers of GFP^+^ leukocytes, F4/80^+^ macrophages, and GFP^+^F4/80^+^ macrophages in the UUO kidneys of *Cd248*^*–/–*^ parabionts although these cell numbers were similar between control kidneys of WT and *Cd248*^*–/–*^ parabionts (Fig. 3c,d), suggesting that loss of CD248 reduced monocyte recruitment to injured kidneys.

Transcripts of M2-biased cytokine genes *Ccl17* and *Ccl22* were downregulated in the macrophages isolated from UUO kidneys of *Cd248*^*–/–*^ mice, whereas M1-biased cytokines and enzymes were not affected (Fig. 3e). In the transwell co-cultures of RAW264.7 macrophages with WT UUO-kidney myofibroblasts, the transcripts of *Nos2*, *Arg1* and *Ccl17* in the macrophages were increased 2- to 3-folds (Fig. 3f). However, no significant increases were observed for any of the three transcripts in macrophages co-cultured with *Cd248*^*–/–*^ UUO-kidney myofibroblasts (Fig. 3f). Moreover, the effect of CD248 on *Ccl17* expression was re-confirmed in bone marrow-derived macrophages (BMDMs) co-cultured with WT or *Cd248*^*–/–*^ myofibroblasts (Fig. 3g). Supplementation with recombinant CD248 (rCD248) increased *Ccl17* expression in BMDMs co-cultured with either WT or *Cd248*^*–/–*^ myofibroblasts (Fig. 3g). Moreover, CCL17 was found to increase transwell migration of RAW264.7 macrophages but the effect was blocked by anti-CCL17 antibody (Supplementary Figure S8a,b), suggesting an autocrine stimulatory effect of CCL17 on macrophage migration. To study the *Ccl17*-expressing cells in the kidneys, we performed in situ hybridization for *Ccl17* and *Adgre1* (Supplementary Figure S9). *Ccl17* and *Adgre1* increased expression in the interstitium of UUO kidneys, and the principal *Ccl17*-expressing cells were *Adgre1*^+^ macrophages. These results demonstrate an influence of CD248 on CCL17 expression of macrophages thereby stimulating the migration of macrophages into injured kidneys.

**Figure 4:**
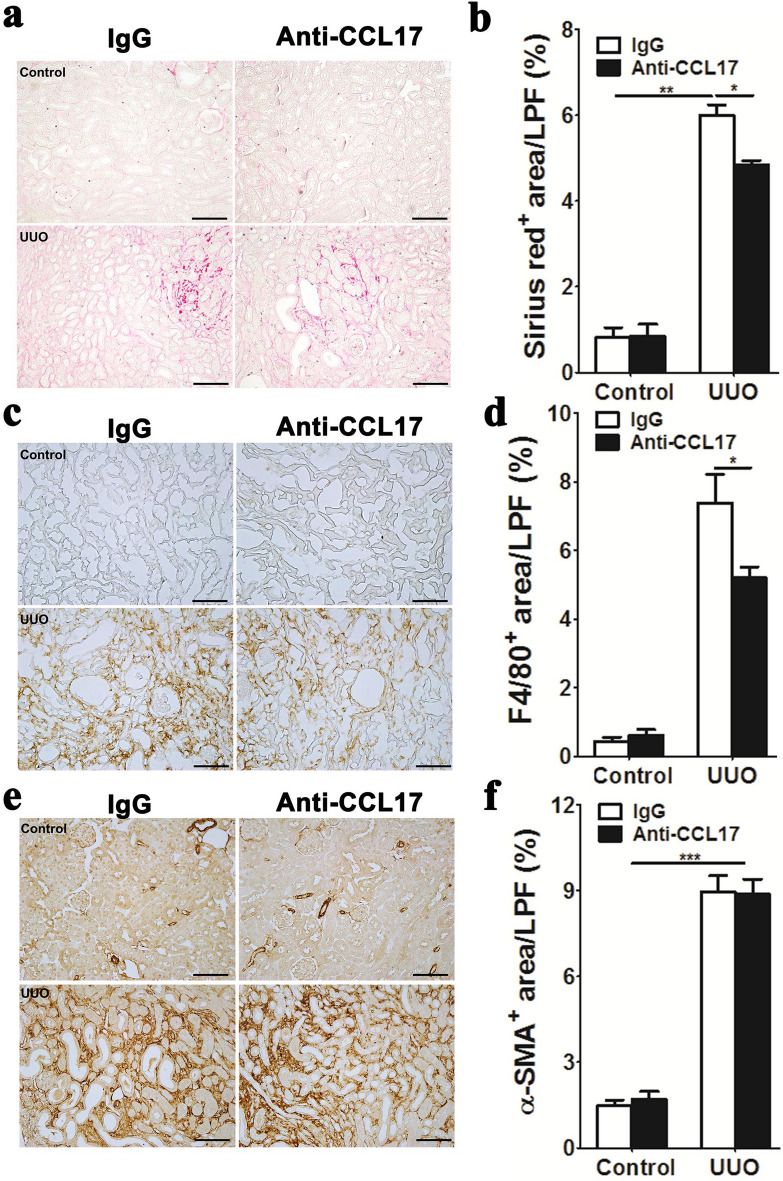
Anti-CCL17 antibody administration attenuated murine obstructive renal fibrosis. (**a**) Representative images of picrosirius red staining in control and UUO kidneys on day 10 after surgery in mice administered with anti-CCL17 antibody or control IgG. (**b**) Quantification of Sirius red^+^ collagen fibrils on LPF images of kidney sections. **(c)** Representative images of F4/80 immunostaining for macrophages in control and UUO kidneys on day 10 after surgery. (**d**) Quantification of F4/80^+^ areas on LPF images of kidney sections. (**e**) Representative images of αSMA immunostaining for myofibroblasts in control and UUO kidneys on day 10 after surgery. (**f**) Quantification of αSMA^+^ areas on LPF images of kidney sections. The original magnification of representative images was × 100 in (**a**,**c**,**e**) and the LPF images of kidney sections were taken at 100 × magnification in (**b**,**d**,**f**). Scale bar, 100 μm. Data are expressed as means ± standard errors of the mean. *n* = 6. *P < 0.05, **P < 0.01 and ***P < 0.001 by one-way ANOVA with post hoc Tukey’s multiple comparisons test.

The pro-fibrotic influence of CCL17 in the UUO model was confirmed with anti-CCL17 antibody administration (Fig. 4). Anti-CCL17 antibody administration attenuated Sirius red^+^ collagen deposition and fibrosis (Fig. 4a,b) as well as F4/80^+^ macrophage accumulation in UUO kidneys (Fig. 4c,d), but not αSMA^+^ myofibroblasts (Fig. 4e,f).

### CD248 interacts specifically with galectin-3 and promotes CCL17 expression in macrophages

**Figure 5:**
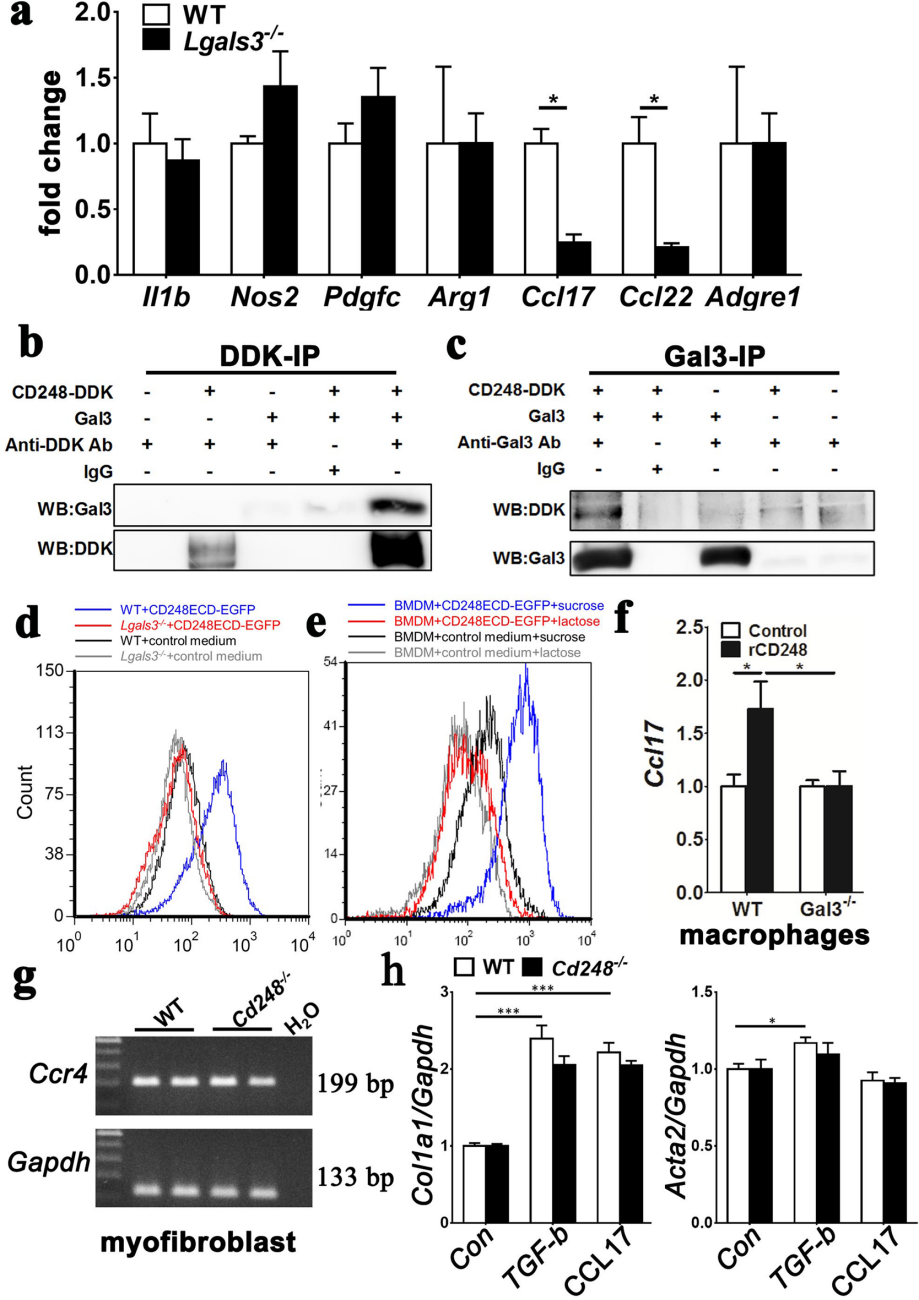
CD248 interacted with galectin-3 to induce CCL17-expressing pro-fibrotic macrophages. (**a**) qPCR of genes encoding cytokines and chemokines in macrophages isolated from D7 UUO kidneys of WT and *Lgals3* knockout (*Lgals3*^*–/–*^) mice. *n* = 5. (**b,c**) HEK293T cells were transfected with and without plasmid DNA expressing galectin-3 or CD248-DDK separately. CD248-DDK (DDK-IP) or galectin-3 (Gal3-IP) was immunoprecipitated from the cell lysates, and then immunoblot analyses of galectin-3 and CD248-DDK were performed. (**d**) Flow cytometry of the binding of CD248 extracellular domain (CD248ECD-EGFP) to WT and *Lgals3*^*–/–*^ BMDMs. Blue and red lines indicate results for WT and *Lgals3*^*–/–*^ BMDMs, respectively, with CD248ECD-EGFP. Black and gray lines indicate results for WT and *Lgals3*^*–/–*^ BMDMs, respectively, with control medium. (**e**) Flow cytometry of CD248ECD-EGFP binding to WT BMDMs in the presence of 25 mM lactose (red) or sucrose (blue). Conditioned medium from HEK293T cells in the presence of lactose (gray) or sucrose (black) was used as control. (**f**) qPCR of *Ccl17* in WT and *Lgals3*^*–/–*^ BMDMs in the absence (control) or presence of 200 ng/ml rCD248 for 48 h. *n* = 6. (**g**) Gel plots of PCR for *Ccr4* and *Gapdh* in D7 UUO-kidney myofibroblasts isolated from WT and *Cd248*^*–/–*^ mice. The reaction cycles for *Ccr4* and *Gapdh* were 30 and 15, respectively. (**h**) qPCR for genes *Col1a1* and *Acta2* in isolated D7 UUO-kidney myofibroblasts after incubation with vehicle (Con), TGF-β1, and CCL17 for 24 h. Data are expressed as means ± standard errors of the mean. *P < 0.05, **P < 0.01 and ***P < 0.001 by unpaired t-test in (**a**) and one-way ANOVA with post hoc Tukey’s multiple comparisons test in (**f**,**h**).

Because galectin-3 plays a significant role in the activation of M2 macrophages and UUO-induced renal fibrosis^28,29^, we were intrigued about the interaction between galectin-3 and CD248 in renal fibrosis. The transcripts of *Lgals3* increased in the UUO kidneys after surgery (Supplemental Figure S10). We confirmed that UUO-induced renal fibrosis was decreased in *Lgals3*^*–/–*^ mice (Supplementary Figure S11). Compared to the expression in the UUO-kidney macrophages of WT mice, UUO-kidney macrophages of *Lgals3*^*–/–*^ mice had reduced expression of *Ccl17* and *Ccl22* while the expression of M1-biased cytokines and enzymes was not different (Fig. 5a), complementing our observations in *Cd248*^*–/–*^ mice. In addition to macrophages, the epithelia of renal collecting ducts were positive for galectin-3 immunostaining (Supplementary Figure S12). To study whether galectin-3 was expressed in renal pericytes or myofibroblasts, we performed in situ hybridization for *Lgals3* and *Pdgfrb* (Supplementary Figure S13). *Pdgfrb* increased expression in interstitium after UUO surgery, but no *Pdgfrb*-expressing cells were positive for *Lgals3*. We therefore studied the possible mechanism of myofibroblast CD248 underlying the promotion of CCL17 expression in macrophages subsequently. In the high-powered field image, the proximity of CD248-galectin-3 could be demonstrated (Supplemental Figure S14). We then expressed CD248-DDK and galectin-3 in separate HEK293T cells for use in co-immunoprecipitation cell lysate assays. Anti-DDK antibody pulled down CD248 in association with galectin-3; reciprocally, anti-galectin-3 antibody pulled down galectin-3-associated CD248 (Fig. 5b,c). CD248 extracellular domain-enhanced GFP fusion protein (CD248ECD-EGFP) bound WT BMDMs but not *Lgals3*^*–/–*^ BMDMs (Fig. 5d). Binding specificity was demonstrated by reversing the binding with the addition of lactose, not sucrose to the reaction (Fig. 5e).

The functionality of the CD248 and galectin-3 interaction was analyzed in assays for *Ccl17* expression. rCD248 upregulated *Ccl17* in WT but not *Lgals3*^*–/–*^ BMDMs (Fig. 5f). Complementing the finding for pro-fibrotic influence of CCL17 in the UUO model (Fig. 4), CCL17 stimulated *Col1a1* expression of UUO-kidney myofibroblasts who expressed C–C motif chemokine receptor 4 (CCR4) (Fig. 5g,h). Both WT and *Cd248*^*-/-*^ myofibroblasts expressed CCR4, and no difference was found in the expression of *Col1a1* stimulated by CCL17 (Fig. 5g,h). No effect of CCL17 on *Acta2* expression was found (Fig. 5h). Taken together, these data indicate that myofibroblast CD248 induced pro-fibrotic phenotype switching of macrophages through galectin-3, and that macrophage CCL17 upregulated collagen expression of myofibroblasts thereby leading to renal fibrosis. Loss of CD248 reduced CCL17 production by macrophages and then collagen production by myofibroblasts.

### Vaccination with *Cd248* cDNA fused with the C fragment of tetanus toxoid attenuates renal fibrosis

**Figure 6:**
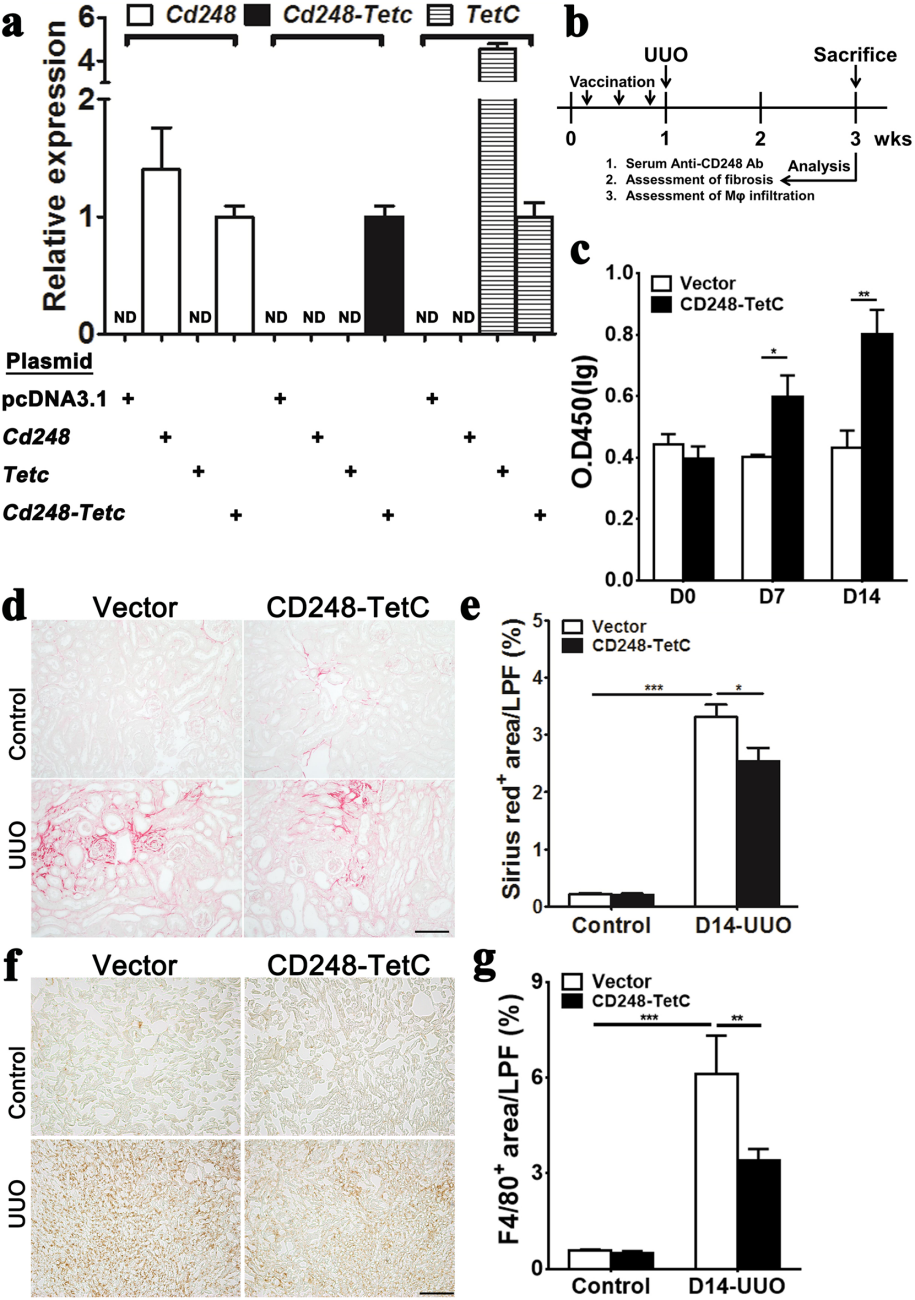
*Cd248* DNA vaccination attenuated renal fibrosis. (**a**) qPCR of *Cd248*, *Cd248-Tetc* and *Tetc* after plasmids were transfected into HEK293T cells. ND: no detection. (**b**) Schema illustrating *Cd-248* DNA vaccination, UUO surgery, and analysis. (**c**) Plasma anti-CD248 antibody was detected by ELISA. (**d**) Representative images of picrosirius red staining in control and D14 UUO kidneys. (**e**) Quantification of Sirius red^+^ collagen fibrils on LPF images of kidney sections. (**f**) Representative images of F4/80^+^ staining in control and D14 UUO kidneys. (**g**) Quantification of F4/80^+^ area on LPF images of kidney sections. The original magnification of representative images was × 100 in (**d**,**f**) and the LPF images of kidney sections were taken at 100× magnification in (**e**,**g**). Scale bar, 100 μm. Data are expressed as means ± standard errors of the mean. *n* = 8–10. *P < 0.05, **P < 0.01 and ***P < 0.001 by unpaired t-test in (**c**) and one-way ANOVA with post hoc Tukey’s multiple comparisons test in (**e**,**g**).

We hypothesized that vaccination with *Cd248* cDNA fused to the C fragment of tetanus toxoid (*Cd248-TT*) would confer adaptive immunity targeting kidney myofibroblasts and thereby attenuate renal fibrosis. To test this possibility, *Cd248–*, *Cd248-TT–*, and *Tetc–*expressing plasmids were transfected into HEK293T cells and their gene expression was confirmed (Fig. 6a). Anti-CD248 antibodies were detected after intra-muscular plasmid DNA vaccination by electroporation (Fig. 6b,c). Picrosirius red staining showed attenuation of renal fibrosis in the UUO kidneys of mice following *Cd248-TT* vaccination (Fig. 6d,e). The increased transcripts of *Col1a1* in the UUO kidneys were also reduced in mice following *Cd248-TT* vaccination (Supplementary Figure S15). Moreover, although F4/80^+^ macrophages accumulated in UUO kidneys, their accumulation was reduced in *Cd248-TT* vaccinated mice, relative to that in control vector vaccinated mice (Fig. 6f,g). Thus, CD248 vaccination attenuated renal fibrosis effectively.

## Discussion

The mechanisms by which macrophages become polarized to a pro-fibrotic phenotype have yet to be fully defined. It may be that a combination of signals from epithelial cells, endothelial cells, myofibroblasts, and perhaps other inflammatory cells trigger phenotype switching in recruited macrophages^14,17,18,30^. The present results are consistent with the notion that signals from myofibroblasts in particular play an important role in enabling fibrosis of tissue tissues. Moreover, we demonstrated, for the first time, that pro-fibrotic phenotype switching of macrophages during tissue fibrosis involves an interaction from CD248 of myofibroblasts.

The early influx of pro-inflammatory monocytes/macrophages into injured organs has been shown to promote apoptosis of injured parenchymal cells and remove cellular debris^14,17,18,20–24^. These pro-inflammatory macrophages switch functionally to a trophic phenotype that suppresses inflammation and promotes cell proliferation and the repair of parenchyma during the resolution phase of acute injury healing^17,20,30–32^. However, when pro-inflammatory macrophages switch to a pro-fibrotic phenotype, they produce growth factors and cytokines that promote myofibroblast proliferation, resulting in tissue fibrosis, especially under conditions of a persistent or recurring injury^14,18,19,21–24,30^. Pro-inflammatory macrophages are believed to be recruited to injured tissue in response to chemoattractants produced by resident dendritic cells/macrophages and injured epithelial/endothelial cells^9,14,17,18,33–35^. Previous studies on progressive tissue fibrosis have focused on the pro-fibrotic effects of macrophages on myofibroblast activation and epithelial/endothelial damage^13–15,19,30,35^. Consistent with previous reports^14,19,21–24,30^, the present results demonstrate myofibroblasts can be activated by CCL17 from macrophages. This finding is in line with previously reported evidence showing that macrophage ablation protects against myofibroblast accumulation and tissue fibrosis, possibly through the amelioration of CCL17-induced activation of myofibroblasts^18,28,36^.

Galectin-3 has been shown crucial for IL-4/IL-13–induced macrophage activation^29^. Selective pharmacological inhibition of extracellular galectin-3 carbohydrate binding has also been shown to block IL-4–induced activation, suggesting that the galectin-3 extracellular domain (ECD) may play a role in the alternative pathway of macrophage activation^29^. Although IL-4/IL-13 is able to switch pro-inflammatory macrophages to arginase-1–, mannose receptor–, and IL-10–expressing macrophages in vitro, similar phenotype switching has been shown to occur independent of IL-4/IL-13 signaling in vivo^17,26^.

In contrast to the indiscernible effect of IL-4/IL-13 signaling on the pro-fibrotic phenotype switching of macrophages in vivo^17,26^, our parabiosis and co-culture experiments showed that myofibroblasts can promote the macrophage recruitment and pro-fibrotic phenotype switching through an interaction of CD248 and galectin-3 during tissue injury and fibrosis. Upon injury, myofibroblasts increased in number and surrounded the recruited pro-inflammatory macrophages. Through the interaction between CD248 and galectin-3, CCL17 was induced in macrophages to activate migration of macrophages and collagen production of myofibroblasts. Conversely, in the absence of CD248, we observed reduced numbers of pro-fibrotic macrophages and reduced expression of CCL17, and these effects were associated with decreased tissue fibrosis. In line with our findings, CCL17, a M2 macrophage-skewed chemokine, shows pro-fibrotic action in tissue fibrosis including lung, skin and peritoneum^18,24,37–39^. Our data showed that more than 80% of pericytes differentiated into αSMA^+^ myofibroblasts on day 14 after UUO surgery and loss of CD248 led to decreased collagen expression rather than cell numbers of myofibroblasts. The proportion of positive αSMA immunostaining in Col1a1-GFP^+^ pericytes on day 14 after UUO surgery was higher than the reported data derived from the dual reporter mouse that expresses red fluorescence protein (RFP) under direction of the *Acta2* enhancer/ promoter (αSMA-RFP) in addition to Col1a1-GFP^40^. We thought this discrepancy was a result of different technique used in the experiments^41^. We would be very interested to know the sensitivity and specificity of immunostaining using αSMA antibody in the αSMA-RFP reporter mouse.

We demonstrated that CD248 bound to WT, but not *Lgals3*^*–/–*^ macrophages and WT, but not *Cd248*^*–/–*^ myofibroblasts induced *Ccl17* expression in macrophages. Moreover, our result of CD248ECD-EGFP binding to galectin-3 on macrophage membranes in a manner that could be inhibited by lactose indicates that this interaction between the CD248-ECD and galectin-3 is specific. Our co-culture experiments showing that both soluble and membrane-bound CD248 affect the pro-fibrotic behavior of macrophages preclude a distinction between membrane-bound and soluble forms of CD248 as the major effector.

Serum levels of CD248 might be a potential evidence to prove the DNA vaccine was working. However, we could not detect consistent serum levels of CD248. We thought that DNA vaccines mainly transfected muscle cells, thereby leading to an enhanced expression in the muscle cells and a concomitant increase in immune responses to the target antigen. Besides, we used CD248-TT DNA plasmid DNA containing full-length *Cd248* cDNA and the C fragment of TT which has been used to elicit antigen-specific immune responses. The antigen might not be released to circulation at a detectable level because the target antigen would be a membrane bound CD248. A similar approach did not show the antigen detected in serum after DNA vaccination and the researchers showed the serum antibody titer in the animal model^42^.

The presently demonstrated effect of CD248 on macrophages represents a novel molecular function in pro-fibrotic macrophage switching and myofibroblast activation. CD248 could potentially be targeted clinically to attenuate tissue fibrosis. Because of its restricted expression in normal tissues and upregulation in chronic fibrotic disease, blockade of CD248 in particular should be prioritized for clinical studies.

## Methods

### Mice

*Cd248*^*lacZ/lacZ*^ mice have been described in detail in our previous study^2^. In brief, the *Cd248* gene was disrupted by replacing its single exon with the *lacZ* reporter gene. The mice were backcrossed to *C57BL/6* mice for more than 10 generations. Mice were bred by mating heterozygous (+ /lacZ) parents. Littermates of the wild type (WT, *Cd248*^+*/*+^), and homozygous knockin (*Cd248*^*lacZ/lacZ*^) mice were used for the experiments. Because homozygous *lacZ* knockin led to homozygous *Cd248* knockout, *Cd248*^*lacZ/lacZ*^ mice were termed *Cd248*^*–/–*^ mice thereafter. *Col1a1-GFP*^*Tg*^ mice were generated and validated as described previously on the *C57BL/6* background; their kidney pericytes and perivascular fibroblasts expressed enhanced GFP^4,11^. *Cd248*^*–/–*^ mice were bred with *Col1a1-GFP*^*Tg*^ mice to generate *Cd248*^*–/–*^;*Col1a1-GFP*^*Tg*^ mice. Littermate *Cd248*^+*/*+^;*Col1a1-GFP*^*Tg*^ mice were used as controls. *Lgals3*^*-/-*^ mice were galectin-3 null; they were developed as described previously and backcrossed to *C57BL/6* mice^43^. *C57BL/6-Tg(CAG-EGFP)1Osb/J* (hereafter referred to as *GFP*^*Tg*^) mice were generated and validated as previously described on the *C57BL/6* background whose cells, with exception of erythrocytes and hair, were green under excitation light^44^. WT *C57BL/6* mice were obtained from The Jackson Laboratory (Stock No: 000664, Bar Harbor, ME). All studies were carried out under protocols approved by our Institutional Animal Care and Use Committee.

### Models of organ fibrosis

A model of progressive renal fibrosis induced by UUO was conducted with male adult (8–12-week-old) mice according to a previously described method^4,11^. Briefly, under anesthesia induced by ketamine/xylazine (100/10 mg/kg body weight intraperitoneally), each left ureter was exposed through flank incision with the mouse in the prone position. The ureter was ligated twice using 4–0 nylon surgical sutures at a level referenced to the lower pole of kidney to ensure that obstruction had a comparable effect in every mouse. The contralateral kidney in the same mouse served as a control. In neutralization experiments, UUO mice were injected with 4 μg of anti-mouse CCL17 (AF529; R&D Systems, Minneapolis, MN) or isotype control immunoglobulin G (IgG) (AB-108-C; R&D Systems) intraperitoneally daily until day 10 after UUO induction^37^.

A model of progressive renal fibrosis induced by uIRI was induced in male adult (8–12-week-old) mice according to a previously described method^4^. In brief, the left kidney of each anesthetized mouse was exposed through a surgical flank incision. A non-traumatic micro-aneurysm clip was placed across the renal artery and vein under a homeothermic blanket system (Stoelting Co., Wood Dale, IL), which contained a rectal thermal probe and a heating pad to maintain the core body temperature at 37 °C. The kidney was confirmed to become dusky, and was replaced in the retroperitoneum for 30 min. The clamps were removed and the return of perfusion to the kidneys was confirmed before wound closure.

A model of progressive peritoneal fibrosis induced by injection of sodium hypochlorite was conducted according to a previously described method^27^. Briefly, injury was induced by intraperitoneal injection of 100 ml/kg body weight normal saline with 0.05% sodium hypochlorite. The peritoneal adhesion score was determined by assigning 1 point for each adhesion present between the abdominal wall and intestine, intestine and intestine, intestine and omentum, omentum and kidney, and kidney and liver, according to a previously described method^27^.

### Parabiosis

Parabiosis was performed in adult (8–12-week-old) mice according to a previously described method^45^. Briefly, anesthetized *GFP*^*Tg*^, WT and *Cd248*^*–/–*^ mice were shaved and a unilateral flank skin incision from the elbow to the knee joint was created in each. The skin edge was sutured with 5.0 prolene to generate *GFP*^*Tg*^-WT and *GFP*^*Tg*^-*Cd248*^*–/–*^ parabiotic mice. Fourteen days after parabiotic surgery, UUO was performed in *GFP*^*Tg*^, WT and *Cd248*^*–/–*^ mice. Mice were sacrificed on day 3 after injury to analyze the recruitment of GFP^+^ cells into injured kidneys of WT and *Cd248*^*–/–*^ mice.

### Tissue preparation and histology

After sacrificed, mouse tissues including the kidneys and peritoneum were collected for pathology, RNA, and protein preparation. Mouse tissues were prepared and stained as described previously^4,11,27^. Primary antibodies against the following proteins were used for immunolabeling: α-smooth muscle actin (αSMA, 5694; Abcam, Cambridge, UK) and Cy3-conjugated αSMA (αSMA-Cy3, C6198; Sigma, St. Louis, MO) for detection of myofibroblasts and vascular smooth muscle cells, Ki-67 (15,580; Abcam) for detection of proliferating cells and F4/80 (MF48000; Thermo Fisher Scientific, Fremont, CA) for detection of macrophages. Fluorescent conjugated affinity purified secondary antibody labeling (Jackson Immunoresearch, West Grove, PA) was performed with co-labeling with 4′,6-diamidino-2-phenylindole (DAPI) and mounting with Vectashield antifade mounting medium (Vector Laboratories, Burlingame, CA). In immunohistochemical analyses, biotin conjugated affinity purified secondary antibody was applied, and cells were then detected by incubation with an avidin–biotin–horseradish peroxidase (HRP) complex and 3, 3′-diaminobenzidine substrate (Sigma). For whole-mount LacZ staining, the liver and abdominal wall were dissected with a microtome (150 μm), incubated in X-gal solution (pH 7.5) at 37 °C for 16 h and then postfixed, as described previously^20^. Tissues were then counterstained with nuclear fast red (Sigma), dehydrated in alcohols, and mounted with permount. Images were captured and processed using a Leica microscope or Zeiss laser capture confocal microscope^4,11,27^. Specific cells in tissue sections were quantified as described previously^4,11,27^. In brief, sections were co-labeled with DAPI, and GFP^+^ cells were identified by blue and green nuclear co-localization; αSMA^+^ and F4/80^+^ cells were identified by positive staining in > 75% of the cell areas immediately surrounding nuclei (detected by DAPI) with Cy3 fluorescence indicative of antigen expression; Ki-67^+^ cells were identified by positive nuclear staining. Specific cells were counted in 10 randomly selected cortical fields between renal capsule and cortico-medullary junction per mouse at 100× magnification. Interstitial fibrosis was quantified in picrosirius red–stained paraffin sections (Picrosirius Red Stain Kit, 24,901; Polysciences, Inc., Warrington, PA). The morphometry of Sirius red^+^ collagen, αSMA^+^ myofibroblasts and F4/80^+^ macrophages was examined in 10 randomly selected cortical fields between renal capsule and cortico-medullary junction per mouse at 100× magnification using the FoveaPro4 program (Reindeer Graphics, Inc., Asheville, NC). Peritoneal membrane thickness was quantified using Masson’s trichrome staining of the peritoneal covering of the liver, as described previously^27^. *Pdgfrb*, *Lgals3*, *Ccl17* and *Adgre1* transcripts were detected on formalin-fixed, paraffin-embedded kidney tissue sections using the RNAscope Multiplex Reagent Kit according to the manufacturer’s instructions (Advanced Cell Diagnostics, Newark, CA).

### Isolation and culture of pericytes and myofibroblasts from kidneys

Details of pericyte and myofibroblast purification and culture from normal and fibrotic kidneys, respectively, have been provided previously^8,11^. Briefly, the kidneys were decapsulated, diced and then incubated at 37 °C for 30 min (normal kidneys) or 1 h (day 7 UUO-damaged kidneys) with liberase (0.5 mg/ml; Roche Applied Science, Indianapolis, IN) and DNase (100 U/ml; Roche Applied Science) in Hank’s balanced salt solution (HBSS; Gibco, Thermo Fisher Scientific). After passage through a 70-µm nylon cell strainer (BD Biosciences, San Jose, CA), an equal volume of HBSS with 10% fetal bovine serum (FBS) was added to the single cell preparation to stop enzyme activity. The mixture was centrifuged at 2000 rpm for 5 min at 4 °C for cell collection. Cells were then resuspended in 5 ml phosphate-buffered saline (PBS) with 1% bovine serum albumin (BSA), and passed through a 40-μm nylon cell strainer (BD Biosciences). Pericytes and myofibroblasts were purified from the single cell suspension by isolating GFP^+^ cells of *Col1a1-GFP*^*Tg*^ mice using FACSAria cell sorting. Then, total RNA was isolated using a RNeasy Mini Kit (Qiagen, Valencia, CA) or cells were cultured in Dulbecco's modified Eagle medium (DMEM) supplemented with 20% FBS. The primary cultured cells collected between passages 4 and 6 were used in this study. Cultured myofibroblasts were stimulated with TGF-β1 (5 ng/ml, R&D Systems), CCL17 (100 ng/ml, R&D Systems), or vehicle control for 24 h. Then, the total RNA was extracted for gene expression analysis.

### Isolation of renal macrophages by magnetic-activated cell sorting

Mouse kidneys were decapsulated, diced and then incubated at 37 °C for 1 h with liberase (0.5 mg/ml; Roche Applied Science) and DNase (100 U/ml; Roche Applied Science) in HBSS. After passage through a 70-µm nylon cell strainer (BD Biosciences), an equal volume of HBSS with 10% FBS was added to the single cell preparation to stop enzyme activity. The mixture was centrifuged at 2000 rpm for 5 min at 4 °C for cell collection. Cells were then resuspended in 5 ml PBS with 1% BSA, and passed through a 40-μm nylon cell strainer (BD Biosciences). After centrifugation, the cells were resuspended in 500 μl 1 × BD IMag™ buffer (BD Biosciences). The cells were incubated in a tube with 50 μl BD IMag™ anti-mouse CD11b particles-DM (1:100; BD Biosciences) at 4 °C for 30 min. After mixing with 500 μl 1 × BD IMag™ buffer, the tube was immediately placed on the BD IMagnet™ and incubated at 4 °C for 6–8 min. With the tube on the BD IMagnet™, the supernatant was carefully aspirated. The tube was then removed from the BD IMagnet™ and 1 × BD IMag™ buffer was added to 1 ml; it was immediately replaced on the BD IMagnet™ and incubated at 4 °C for 4–6 min. This process was repeated twice. After a final wash, the total RNA of CD11b^+^ macrophages were isolated using a RNeasy Mini Kit (Qiagen, Valencia, CA).

### Bone marrow–derived macrophage culture

Bone marrow was obtained from the femoral bone and cultured in DMEM/F12 medium supplemented with 10% FBS and 20% L929 conditioned medium, according to a previously described method^20^. After 5 days of culture at 37 °C, mature BMDMs were collected for further experiments.

### Transwell migration assay

RAW264.7 cells were incubated for 2 h at 37 °C in starvation medium and subsequently adjusted to 8 × 10^5^ cells/ml. Lower chambers of Costar Transwell plates (5 μm pores size, 3421; Corning, NY) were filled with 0.6 ml of starvation media with or without supplementation of 500 ng/ml recombinant murine CCL17 (529-TR/CF; R&D Systems). The CCL17 neutralizing antibody (AF529; R&D Systems) and isotype control IgG (AB-108-C; R&D Systems) were used at 5 μg/ml. Upper chambers were loaded with 0.1 ml of the cell suspension (8 × 10^4^ cells). After allowing the cells to migrate for 6 h at 37 °C, the upper chambers were fixed in 4% paraformaldehyde, washed and stained with DAPI. The migrated cells were quantified by ImageJ in 10 randomly selected fields per membrane at 100× magnification. Each experiment was performed in triplicate.

### In vitro polarization of macrophages

RAW264.7 cells (5 × 10^5^) were cultured in Transwell plates (0.4 μm pores size, 3413; Corning, NY) and then primed with LPS (100 ng/ml; Sigma) and IFNγ (100 U/ml; PeproTech, Rocky Hill, NJ) for 24 h. After changing to fresh medium, 1 × 10^5^ kidney myofibroblasts from WT and *Cd248*^*–/–*^ mice cultured in Transwell inserts were placed into the wells of microplates. After co-culturing for 48 h, total RNA of RAW264.7 cells was extracted for analysis of M1- and M2-skewed chemokines and receptors.

BMDMs (5 × 10^5^) were cultured in 24-well microplates and then primed with LPS and IFNγ for 24 h. After changing to fresh medium, 1 × 10^5^ myofibroblasts from WT and *Cd248*^*–/–*^ mice were added to the wells. Then, 50 ng/ml rCD248 proteins (R&D Systems) or vehicle was added. After co-culturing for 48 h, the BMDMs were isolated using anti-mouse CD11b particles-DM and magnetic-activated cell sorting (BD Biosciences) for total RNA extraction and then analysis of M1- and M2-skewed chemokines and receptors.

In separate experiments, BMDMs (5 × 10^5^) were treated with 200 ng/ml rCD248 or vehicle for 48 h after priming with LPS and IFNγ. The total RNA of BMDMs was extracted for gene expression analysis.

### Polymerase chain reaction

The purity of total RNA extracted using a RNeasy Mini Kit (for cell samples isolated from in vivo model) or TRIzol (for in vitro cell samples or organs) was determined by A260 to A280. cDNA was synthesized using oligo(dT) and random primers. Quantitative polymerase chain reaction (qPCR) was performed using a previously described method^4^. The specific primer pairs used in qPCR were listed in Supplementary Table S1.

### Immunoprecipitation and immunoblot analysis

HEK293T cells were transfected with plasmid pCMV6-*Cd248*-DDK (MR210537; OriGene Technologies, Inc., Rockville, MD) or pCMV6-*Lgals3* (MC208879; OriGene Technologies, Inc.) by PolyJet reagent (SignaGen Laboratories, Rockville, MD). Total cellular protein was extracted using NP-40 lysis buffer [50 mM HEPES (pH 7.4), 150 nM NaCl, 1% NP-40, 1% protease inhibitor cocktail (Sigma)] and centrifuged at 13,000 rpm for 20 min according to our previously described method^46^. In total, 1 mg lysate from transfected or non-transfected cells (CD248-DKK + galectin-3, CD248-DKK or galectin-3 alone and non-transfected control) was incubated on a shaker at 4 °C overnight. Then, samples were precleared by incubation with Dynabeads Protein G (Thermo Fisher Scientific) and centrifuged at 4,000 rpm for 5 min. Supernatants were collected and incubated with 2 ug anti-DDK (OriGene Technologies, Inc.), anti-galectin-3 (CEDARLANE, Burlington, Canada) and control IgG antibodies, respectively, for 1 h and then incubated with Dynabeads Protein G on a shaker at 4 °C overnight. Elution of immunoprecipitants was performed by heating at 95 °C for 5 min. Immunoblot analysis was performed using a previously described method^8^.

### Galectin-3 and CD248ECD-EGFP binding assay on macrophages

To generate CD248ECD-EGFP fusion protein, the ECD of *Cd248* and *EGFP* were amplified from pCMV-Cd248 and pEGFP-C3 (Takara Bio Inc., Shiga, Japan) and then cloned into pSecTag2/HygroB plasmid (Thermo Fisher Scientific). HEK293T cells were transfected with pSecTag2-*Cd248ECD-EGFP* plasmid, and the culture medium was then changed to DMEM/F12 medium containing ITS supplement (Sigma). After 3 days of culture, the medium was collected and concentrated by an Amicon Ultra-15 Centrifugal Filter (Merck, Temecula, CA). For the binding assay, 100 μl CD248ECD-EGFP or control conditional medium was added to 1 × 10^5^ WT or *Lgals3*^*-/-*^ BMDMs and cultured at 37 °C overnight. For lactose-inhibitable manner, WT BMDMs were incubated with CD248ECD-EGFP or control conditional medium in the presence of 25 mM lactose or sucrose overnight^43^. Cells were harvested and EGFP fluorescence was detected by flow cytometry.

### Fluorescence-activated cell sorting analysis

FACS was performed using FACSCalibur and Fortessa (BD Bioscience), according to a previously described method^14^. Data were analyzed using FCS Express software (De Novo Software, Glendale, CA).

### DNA vaccine, immunization procedures and antibody detection

The vaccine plasmid was constructed according to a previously described method^42^. Briefly, mouse *Cd248* full-length cDNA was ligated to the minimal domain of the C fragment of tetanus toxoid, a gift from Dr. Gordan Dougan^46^. The cDNA construct was cloned into the pCDNA3.1 vector and the sequence was confirmed. DNA immunization was performed according to a previously described method^47^. In brief, 50 μg DNA was injected into mouse leg muscle and immediately electroporated using a NEPA21 Super Electroporator (Nepa Gene Co., Chiba, Japan). Two pulses of electroporation at 100 mV for 200 ms were performed. Anti-CD248 antibodies in serum were detected by enzyme-linked immunosorbent assay. Serum samples were 10 × diluted and added to 96 wells containing 5 ug/ml rCD248 (R&D Systems), then incubated at 4 °C overnight. Anti-mouse IgG-HRP and then 3,3′,5,5′-tetramethylbenzidine were used to detect the reaction. Finally, the optical density at 450 nm was read.

### Statistical analysis

Data were expressed as means ± standard errors of the mean. Unpaired Student’s t-test was used to compare two different groups. One-way ANOVA with post hoc Tukey’s multiple comparisons test was used for the comparison between each group. A *P* value < 0.05 was considered significant. Statistical analyses were carried out using the GraphPad Prism software (GraphPad Software, La Jolla, CA).

### Study approval

All animal studies were carried out under a protocol approved by Institutional Animal Care and Use Committee, National Taiwan University College of Medicine and College of Public Health. The approval numbers were 20150255 and 20190148. All methods were performed in accordance with the relevant guidelines and regulations.

## Supplementary information

Supplementary file1
