# Supplementary material for: Targeting fibroblast CD248 attenuates CCL17-expressing macrophages and tissue fibrosis
Source: Sci Rep. 2020 Oct 8;10:16772. doi: 10.1038/s41598-020-73194-x (PMC7544830; doi:10.1038/s41598-020-73194-x)
Supplement: Supplementary file 1 — Supplementary file1 [file 41598_2020_73194_MOESM1_ESM.pdf]

Pai et al. Supplementary information

Targeting Fibroblast CD248 Attenuates CCL17-expressing Macrophages and Tissue Fibrosis

Chen-Hsueh Pai, Shu-Rung Lin, Chia-Hao Liu, Szu-Yu Pan, Hao Hsu, Yi-Ting Chen, Ching-Tzu Yen, I-Shing Yu, Hua-Lin Wu, Shuei-Liong Lin, Shu-Wha Lin

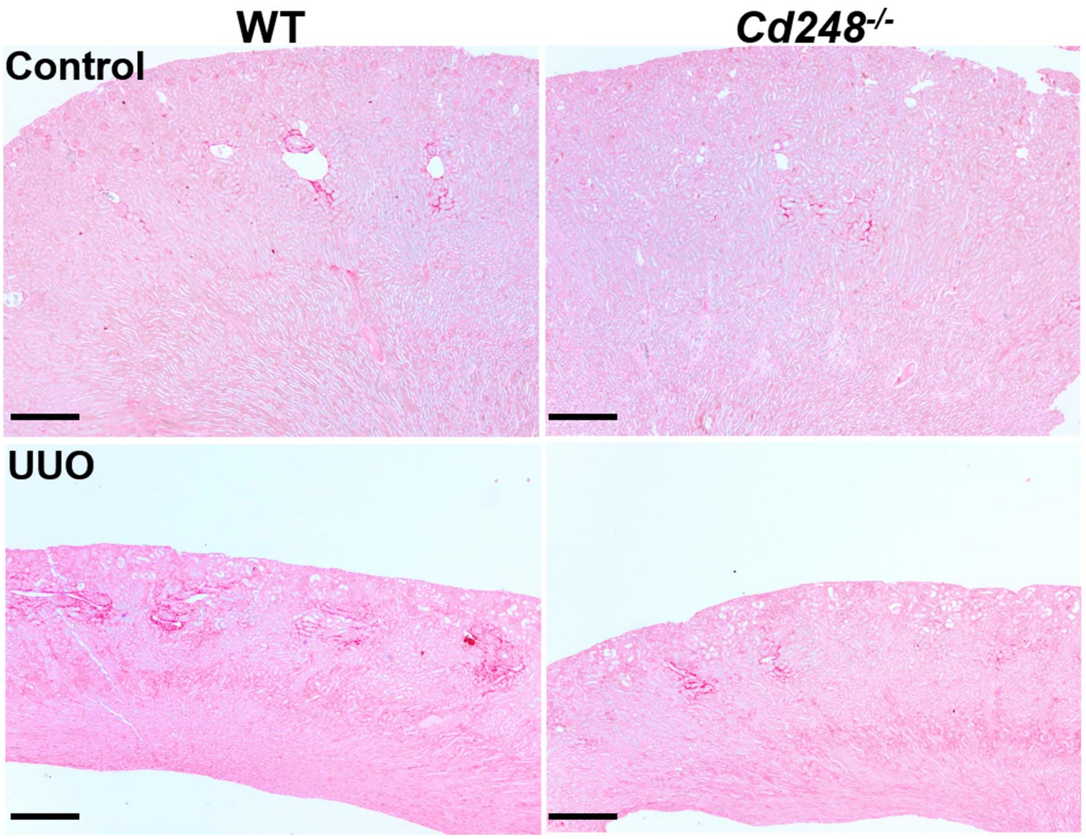

Supplementary Figure S1. Representative images of picrosirius red staining in the kidneys of wild type (WT) and *Cd248* knockout (*Cd248*$^{-/-}$) mice before (control) and on day 14 after unilateral ureteral obstruction (UUO) surgery. Original magnification, ×40. Scale bar, 1 mm.

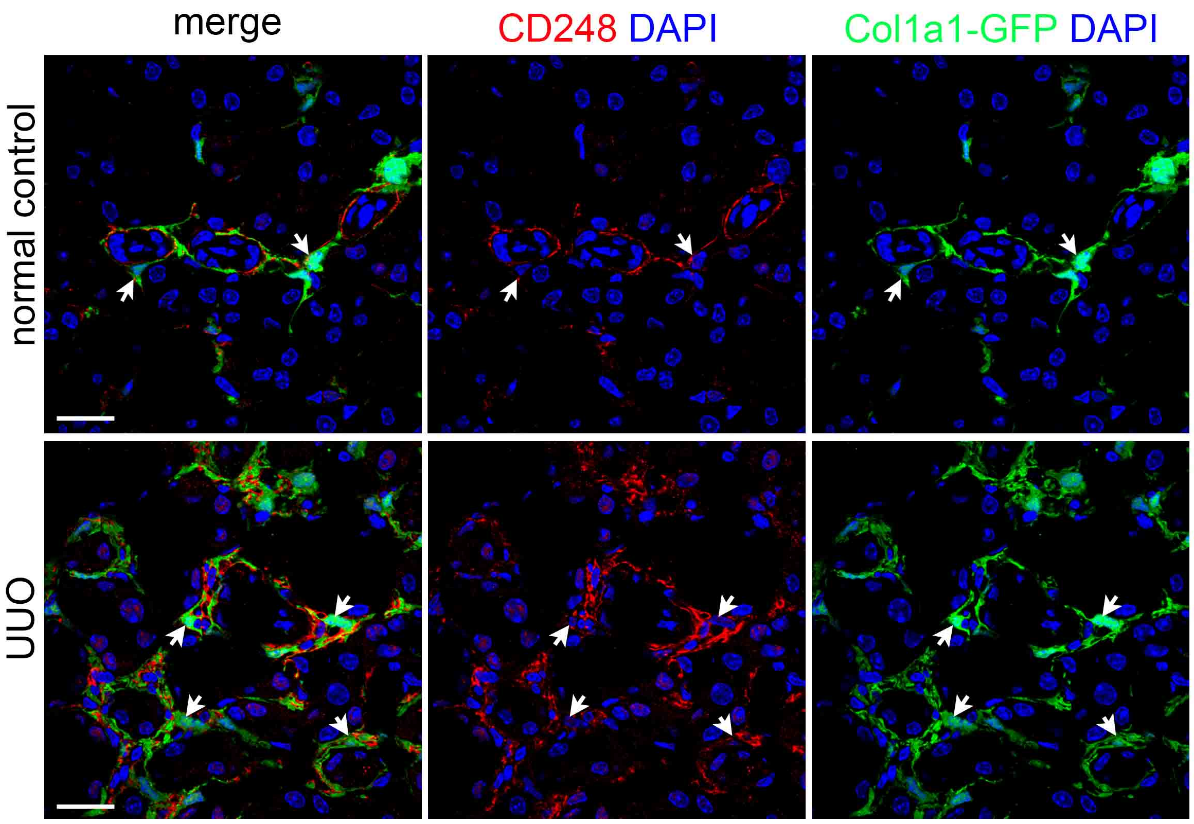

Supplementary Figure S2. CD248 was expressed in pericytes and myofibroblasts of control and fibrotic kidneys of transgenic *Col1a1-GFP* mice, respectively. Fibrotic kidney was induced by UUO surgery and studied on day 7 after surgery. Col1a1-GFP^{+} cells represented pericytes and myofibroblasts of normal and fibrotic kidneys, respectively. CD248^{+}Col1a1-GFP^{+} cells were indicated by arrows. Original magnification, ×630. Scale bar, 50 μm.

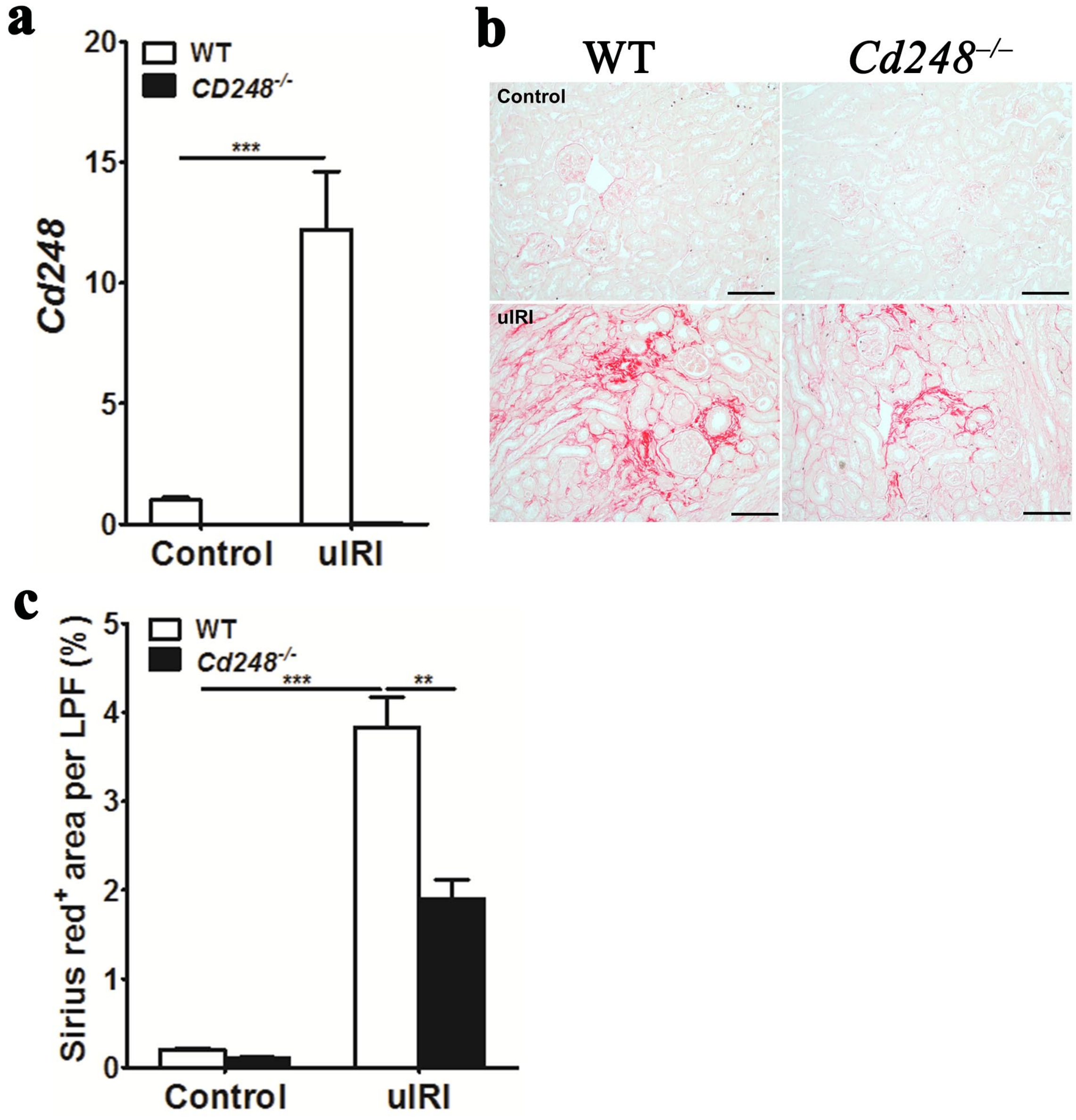

Supplementary Figure S3. *Cd248* disruption attenuated murine ischemic renal fibrosis. **(a)** Quantitative polymerase chain reaction (qPCR) of renal *Cd248* in contralateral (control) and unilateral ischemia/reperfusion injury (uIRI) kidneys of WT and *Cd248*$^{-/-}$ mice on day 14 after surgery. The expression was normalized by *Gapdh* and then compared with that of WT control. *n*=5. **(b)** Representative images of picrosirius red staining in control and uIRI kidneys. Original magnification, ×100. Scale bar, 100 μm. **(c)** Quantification of Sirius red^{+} collagen fibrils on low-powered field (LPF) images of kidney sections taken at 100× magnification. *n*=5. Data are

expressed as means $\pm$ standard errors of the mean. $^{**}P<0.01$, $^{***}P<0.001$ by one-way ANOVA with post hoc Tukey's multiple comparisons test.

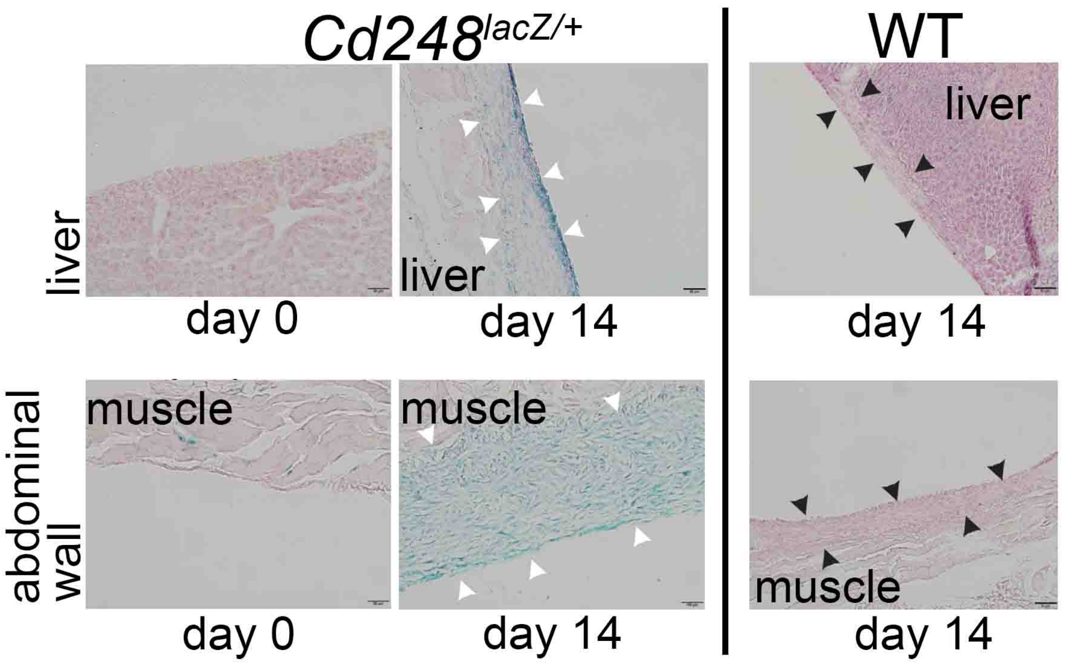

Supplementary Figure S4. *Cd248*-expressing myofibroblasts accumulated in peritoneum with fibrosis induced by peritoneal injection of sodium hypochlorite. X-gal staining images (magnification, ×200) showing increased numbers of *Cd248*-expressing cells within the thickened peritoneum overlying the liver (upper panel) and abdominal wall muscle (lower panel) (indicated by arrowheads) on day 14 after hypochlorite injury. Scale bar, 50 μm.

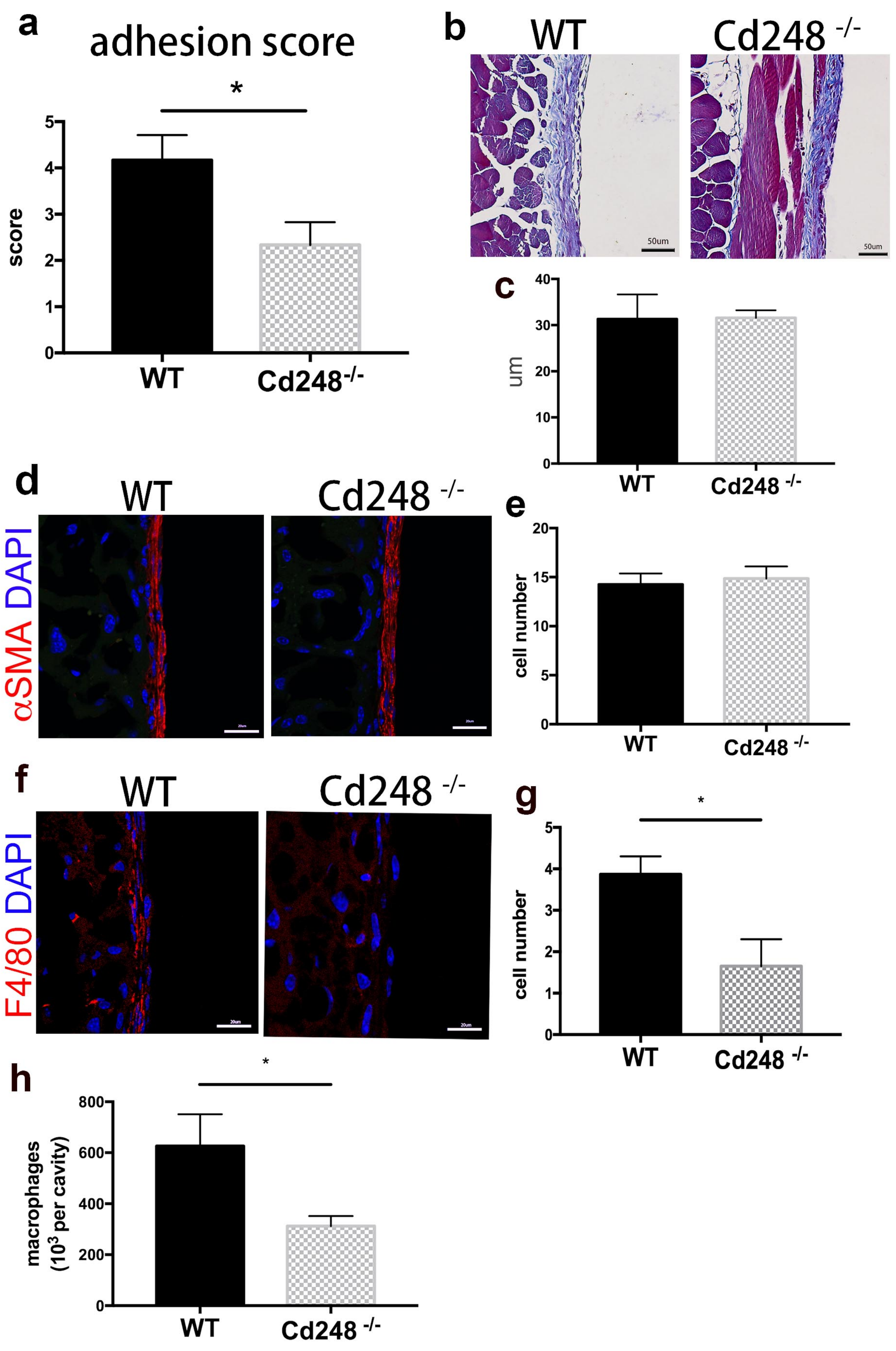
a
adhesion score
*
score
5
4
3
2
1
0
WT
Cd248-/-
b
WT
Cd248 -/-
50um
50um
c
40
30
20
10
0
um
WT
Cd248 -/-
d
WT
Cd248 -/-
αSMA DAPI
e
20
15
10
5
0
cell number
WT
Cd248 -/-
f
WT
Cd248 -/-
F4/80 DAPI
g
*
5
4
3
2
1
0
cell number
WT
Cd248 -/-
h
*
800
600
400
200
0
macrophages
(10³ per cavity)
WT
Cd248 -/-

Supplementary Figure S5. *Cd248* disruption attenuated the murine model of peritoneal fibrosis. **(a)** Graph showing peritoneal adhesion induced by sodium hypochlorite. **(b, c)** Images (magnification, ×200) and graph (membrane thickness, μm) showing the extent of Masson's trichrome blue/green–stained fibrotic peritoneal membrane overlying the liver. Scale bar, 50 μm. **(d, e)** Images (magnification, ×630) and graph (cell number/section) showing the numbers of αSMA^{+} myofibroblasts in fibrotic peritoneum. Scale bar, 20 μm. **(f, g)** Images (magnification, ×630) and graph (cell number/section) showing the numbers of $F4/80^{+}$ macrophages in fibrotic peritoneum. Scale bar, 20 μm. **(h)** Graph showing the number of $CD11b^{+}F4/80^{+}$ macrophages/peritoneal cavity, assessed by flow cytometry. Data in graphs are expressed as means ± standard errors of the mean. *n*=5. **P*<0.05 by two-tailed unpaired t-test.

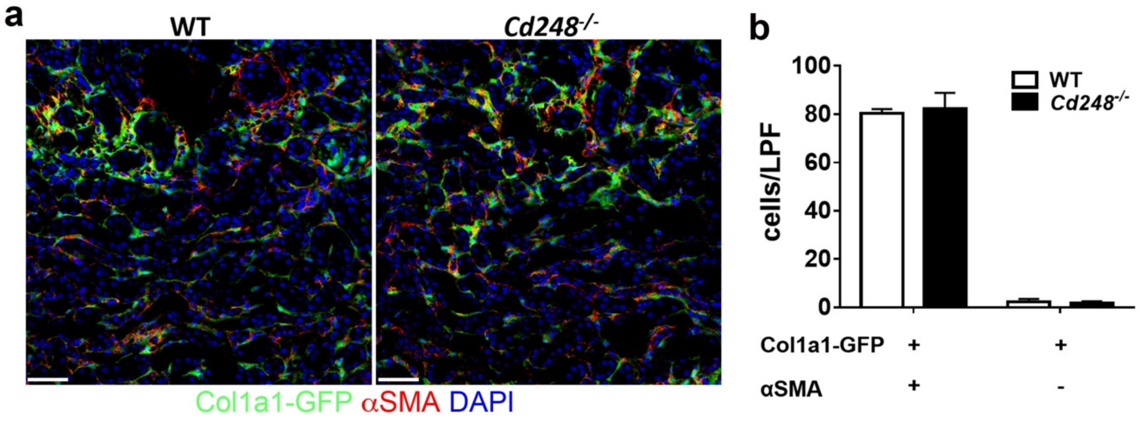

Supplementary Figure S6. *Cd248* disruption did not affect the cell numbers of kidney pericytes and myofibroblasts. **(a)** Representative images of αSMA immunostaining in *Col1a1-GFP*^{Tg} mice with or without *Cd248* knockout before (control) or on day 14 after UUO surgery. Original magnification, ×200. Scale bar, 50 µm. **(b)** Quantification of αSMA^{+}Col1a1-GFP^{+} myofibroblasts and αSMA^{-}Col1a1-GFP^{+} pericytes on LPF images of kidney sections taken at taken at x200 magnification. *n*=6. Data are expressed as means $\pm$ standard errors of the mean. No difference was detected between WT and *Cd248*$^{-/-}$ mice.

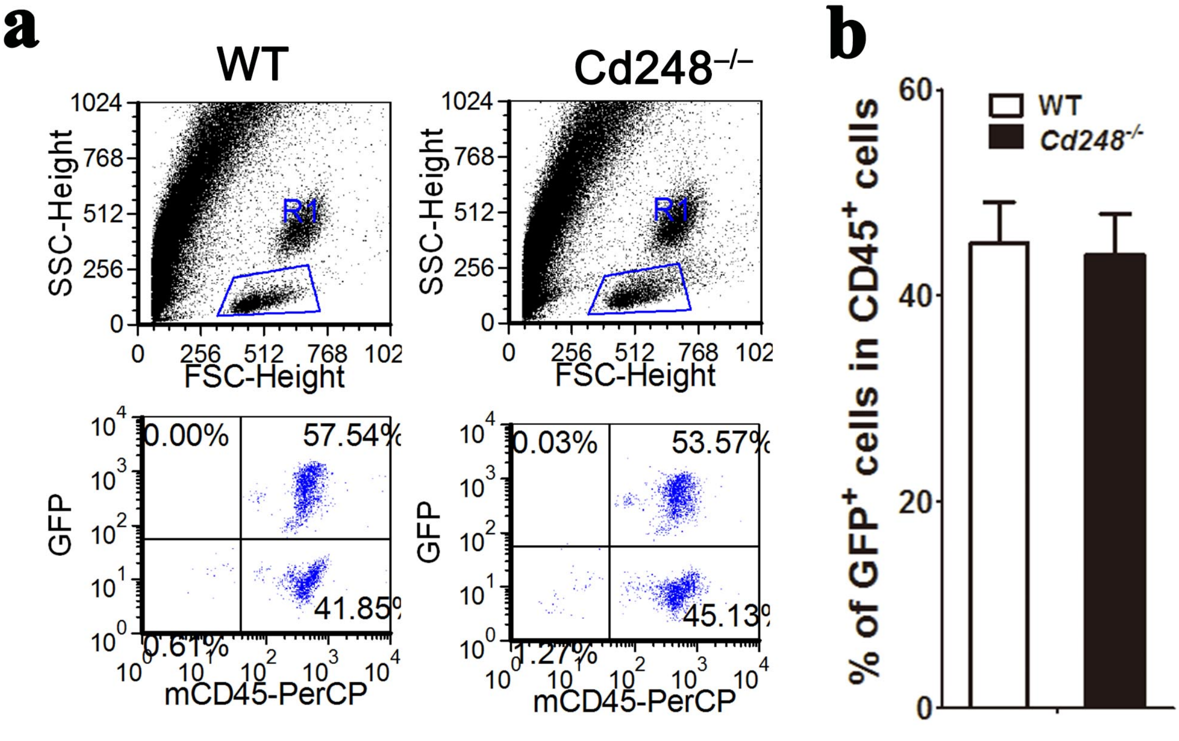

Supplementary Figure S7. Development of cross-circulation by parabiosis. **(a)** Representative flow cytometric plots showing green fluorescent protein (GFP)$^+$ cells in CD45$^+$ peripheral blood mononuclear cells (PBMCs) of WT and *Cd248*$^{-/-}$ parabionts 14 days after parabiosis with *GFP* transgenic mice. **(b)** Blood chimerism, determined by analysis of the percentage of partner-derived GFP$^+$ cells in CD45$^+$ PBMCs of WT and *Cd248*$^{-/-}$ parabionts. *n*=7–9. Data are expressed as means ± standard errors of the mean. No difference was detected.

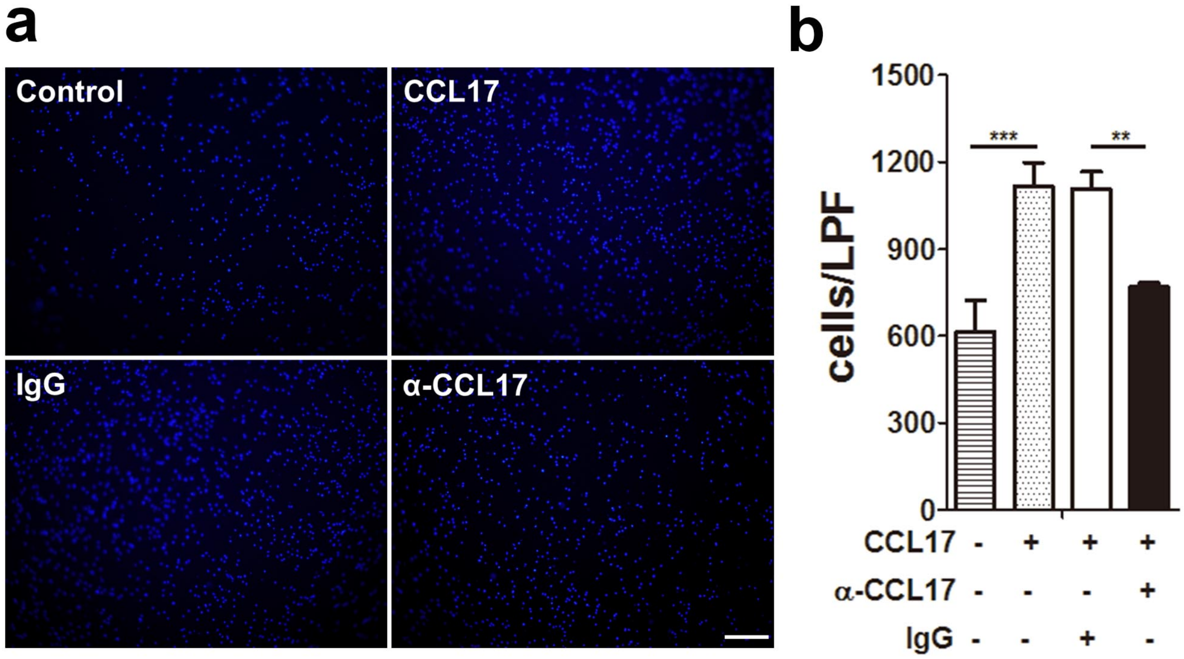

Supplementary Figure S8. CCL17 increased macrophage migration. (a) Representative images of DAPI (Blue) for macrophages (RAW264.7 cells) in Transwell migration assays. Experimental group: Control, medium only; CCL17, 500 ng/ml CCL17; IgG, CCL17 (500 ng/ml) + IgG control (5 μg/ml); and α-CCL17, CCL17 (500 ng/ml) + anti-CCL17 antibody (5 ug/ml). Original magnification, ×100. Scale bar, 100 μm. **(b)** Quantification of $DAPI^+$ cells on LPF images taken at 100× magnification. n=3. Data are expressed as means ± standard errors of the mean. **P<0.01 and ***P<0.001 by one-way ANOVA with post hoc Tukey's multiple comparisons test.

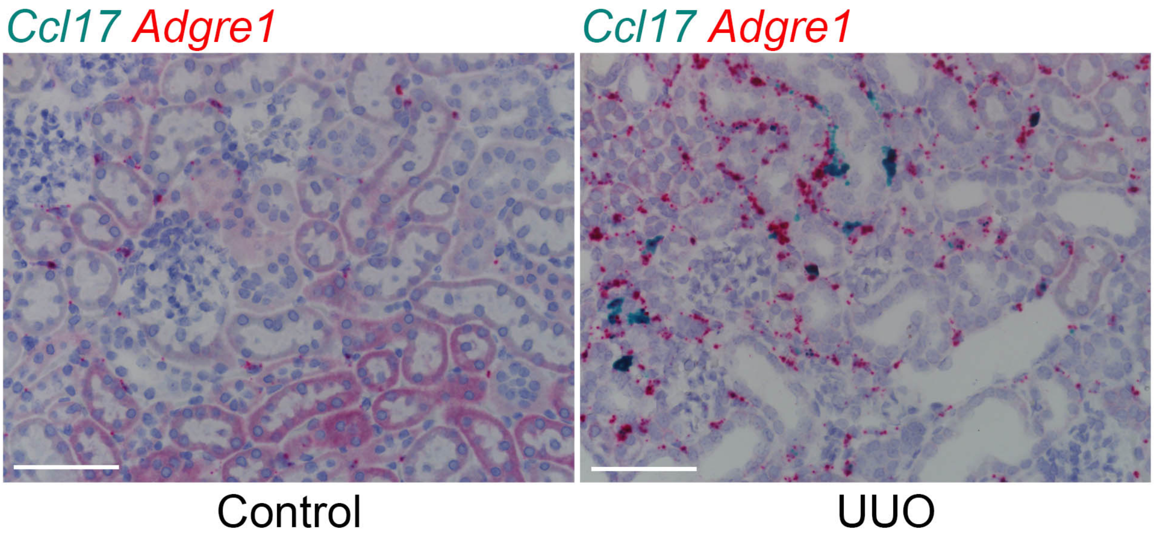

Supplementary Figure S9. *Adgre1*$^+$ macrophages expressed *Ccl17*. Representative images showing the in situ hybridization for *Adgre1* and *Ccl17* which encoded F4/80 and CCL17, respectively. Original magnification, ×400. Scale bar, 100 µm.

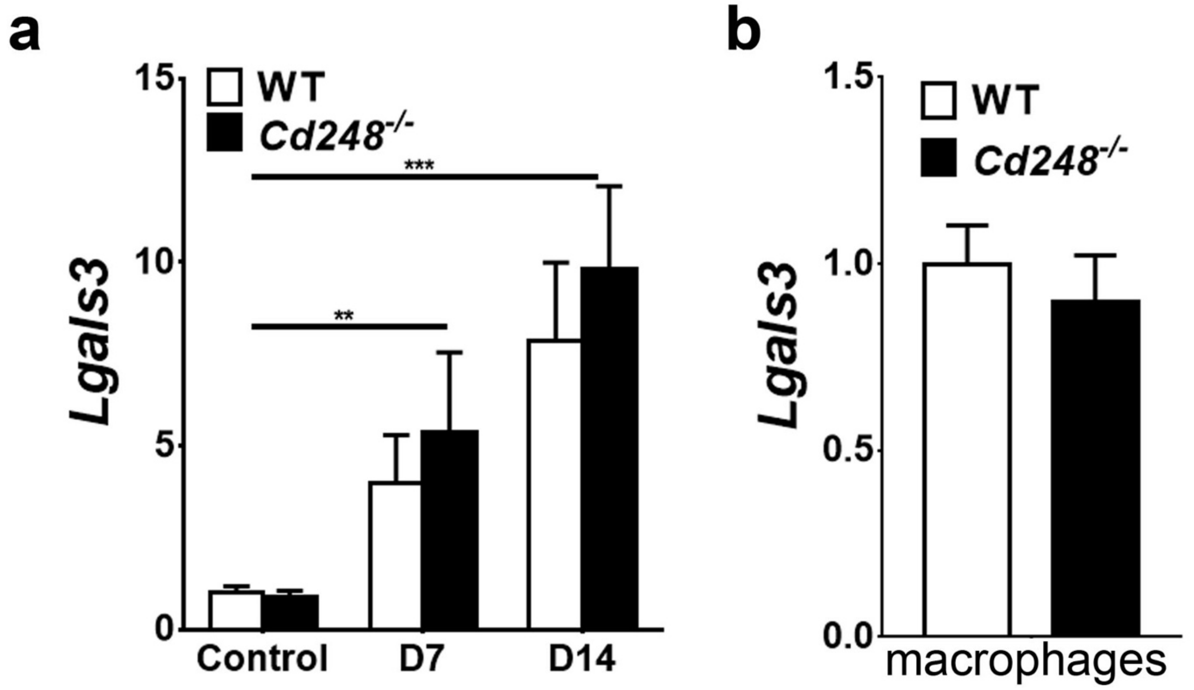

Supplementary Figure S10. *Cd248* disruption did not affect *Lgals3* expression. **(a)** The time course qPCR of renal *Lgals3* in WT and *Cd248*$^{-/-}$ mice after UUO surgery. *n*=5. Control, D7 and D14 indicated the kidneys before and on day 7, day 14 after UUO surgery, respectively. **(b)** qPCR of *Lgals*3 in the macrophages (Mϕ) isolated from UUO kidneys of WT and Cd248$^{-/-}$ mice on day 7 after surgery. *n*=4. Data are expressed as means ± standard errors of the mean. ***P*<0.01 and ****P*<0.001 by one-way ANOVA with post hoc Tukey's multiple comparisons test.

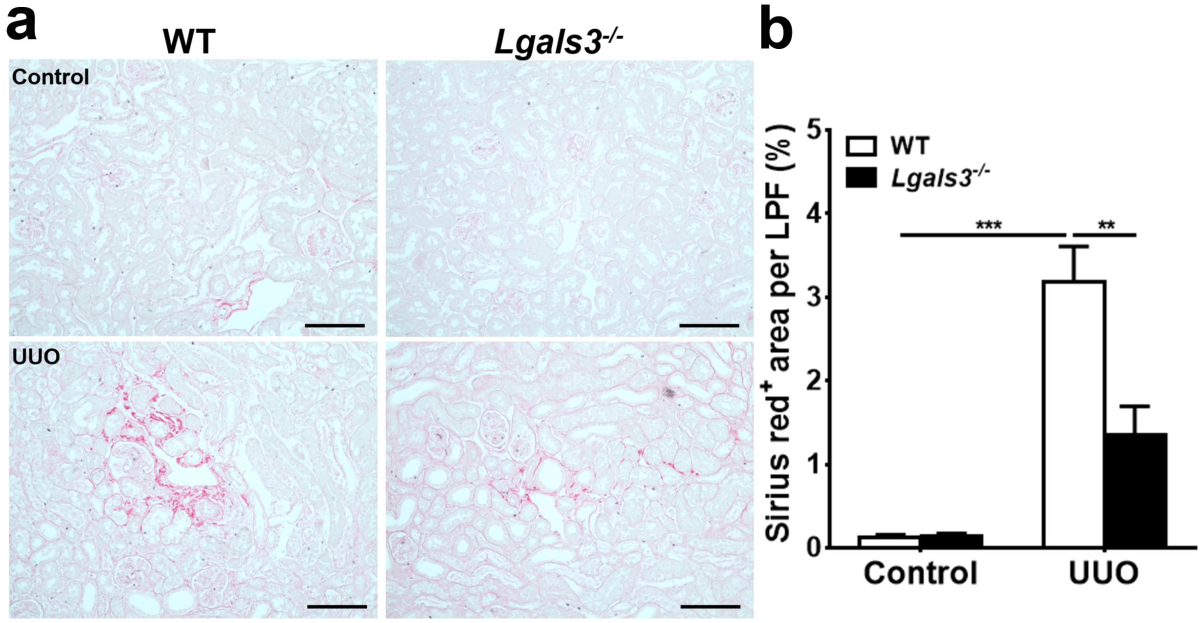

Supplementary Figure S11. *Lgals3* disruption attenuated murine obstructive renal fibrosis. **(a)** Representative images of picrosirius red staining in control and UUO kidneys of WT and *Lgals3*$^{-/-}$ mice on day 14 after surgery. Original magnification, ×100. Scale bar, 100 µm. **(b)** Quantification of Sirius red^{+} collagen fibrils on LPF images of kidney sections taken at x100 magnification. *n*=5. Data are expressed as means $\pm$ standard errors of the mean. ***P*<0.01, ****P*<0.001 by one-way ANOVA with post hoc Tukey's multiple comparisons test.

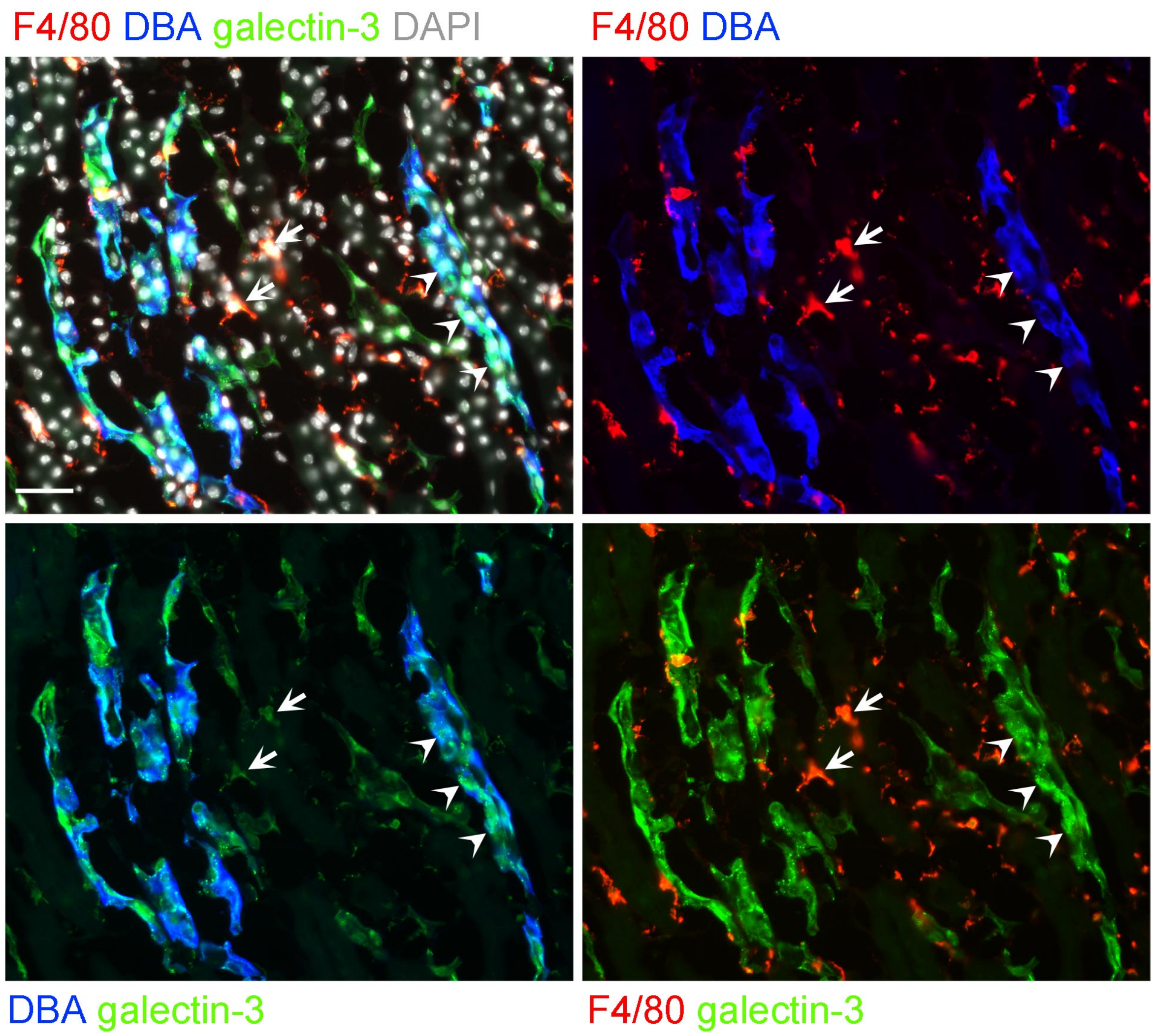

Supplementary Figure S12. Galectin-3 was expressed in epithelia of collecting ducts and macrophages. Epithelia of collecting ducts and macrophages were identified by Dolichos Biflorus Agglutinin (DBA) and F4/80, respectively. Galectin-3^{+}DBA^{+} epithelia of collecting ducts and Galectin-3^{+}F4/80^{+} macrophages were indicated by arrowheads and arrows, respectively. Original magnification, ×400. Scale bar, 100 μm.

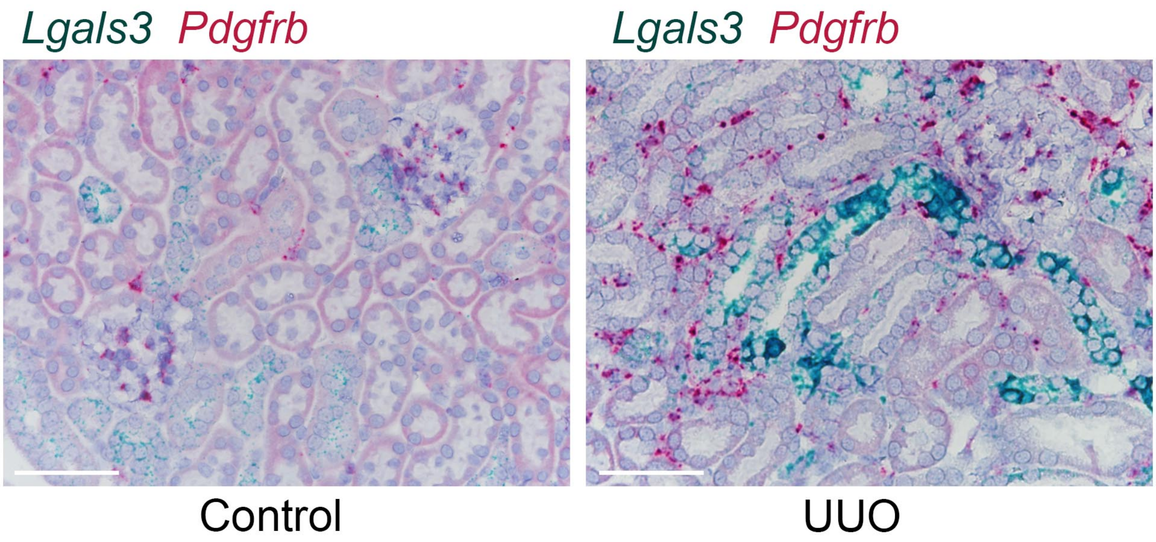

Supplementary Figure S13. *Pdgfrb*$^{+}$ pericytes and myofibroblasts did not express *Lgals3*. Representative images showed the in situ hybridization for *Pdgfrb* and *Lgals3* which encoded platelet-derived growth factor-β and galectin-3, respectively. Original magnification, ×400. Scale bar, 100 µm.

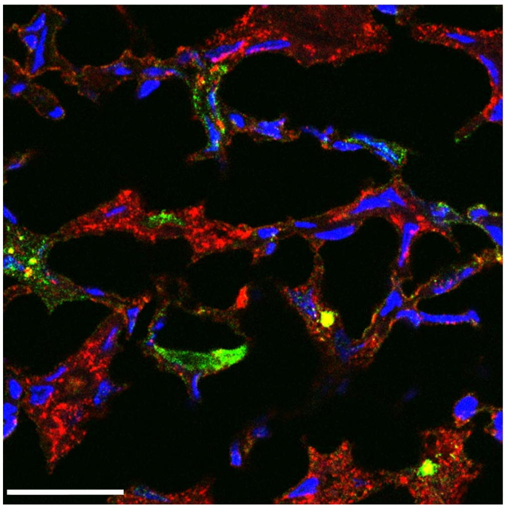

Supplementary Figure S14. The proximity of galectin-3 and CD248 in the injured kidney. Representative image showing the immunostaining of galectin-3 (green) and CD248 (red) in UUO kidneys on day 7 after surgery. Original magnification, ×630. Scale bar, 25 μm.

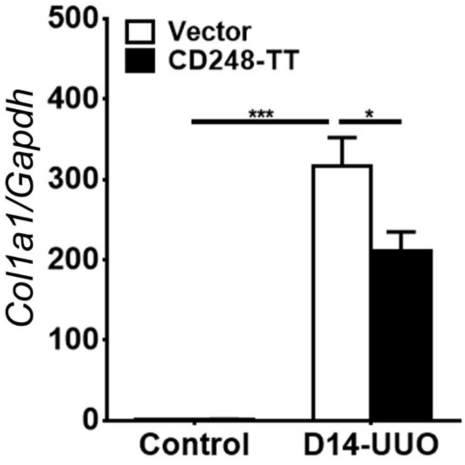

Supplementary Figure S15. *Cd248* DNA vaccination attenuated *Col1a1* gene expression in the UUO kidneys. qPCR of renal *Col1a1* in the control and day 14 UUO kidneys. *Col1a1* expression was normalized by *Gapdh*. n=8-10. Data are expressed as means ± standard errors of the mean. *P<0.05, ***P<0.001 by one-way ANOVA with post hoc Tukey's multiple comparisons test.

Supplementary Table S1. Primer sequences used in quantitative polymerase chain reaction.

Gene		Sequences
mGapdh	Forward	CTG GAG AAA CCT GCC AAG TA
	Reverse	AAG AGT GGG AGT TGC TGT TG
hGapdh	Forward	AGG GCT GCT TTT AAC TCT GGT
	Reverse	CCC CAC TTG ATT TTG GAG GGA
Tgfb1	Forward	GGA CTC TCC ACC TGC AAG AC
	Reverse	GAC TGG CGA GCC TTA GTT TG
Arg1	Forward	GCT GTC TTC CCA AGA GTT GGG
	Reverse	ATG GAA GAG ACC TTC AGC TAC
Ccl17	Forward	AGT GGA GTG TTC CAG GGA TG
	Reverse	CTG GTC ACA GGC CGT TTT AT
Ccl22	Forward	GTG GAA GAC AGT ATC TGC TGC C
	Reverse	AGG CTT GCG GCA GGA TTT TGA G
Pdgfc	Forward	GAG TCC AAC CTG AGC AGC AAG T
	Reverse	GAA ACT TCG GGC TGT GGA TGC T
Nos2	Forward	CAG CTG GGC TGT ACA AAC CTT
	Reverse	CAT TGG AAG TGA AGC GGT TCG
Il1b	Forward	CCC AAG CAA TAC CCA AAG AA
	Reverse	GCT TGT GCT CTG CTT GTG AG
Ccl3/Mip1a	Forward	CGG AAG ATT CCA CGC CAA TTC
	Reverse	GGT GAG GAA CGT GTC CTG AAG
Cxcl2/Mip2a	Forward	CTC TCA AGG GCG GTC AAA AAG TT

	Reverse	TCA GAC AGC GAG GCA CAT CAG GTA
Cd248	Forward	CTG CCA CTC GAC CCA CAC TA
	Reverse	GGT GGG CGT GTA GCT GAA AT
Col1a1	Forward	CTG GTG AAC AGG GTG TTC CT
	Reverse	AGA ACC ATC AGC ACC TTT GG
TetC	Forward	CTT CTC TGG GTC TGG TTG GT
	Reverse	GGT CGG AAC GAA GTA CCA GT
Cd248-TT	Forward	CAA GAG CTC AAC AGA ACC CA
	Reverse	GTT GTC GAC CCA ACA ATC AA
Lgals3	Forward	TTG AAG CTG ACC ACT TCA AGG TT
	Reverse	AGG TTC TTC ATC CGA TGG TTG T
Ccr4	Forward	TCT ACA GCG GCA TCT TCT TCA T
	Reverse	CAG TAC GTG TGG TTG TGC TCT G

Uncut gels/plots for Fig. 5b, 5c, 5g

Fig. 5b α-DDK-IP; WB:α-Gal3

CD248-DDK	-	+	-	+	+
Gal3	-	-	+	+	+
Anti-DDK Ab	+	+	+	-	+
IgG	-	-	-	+	-

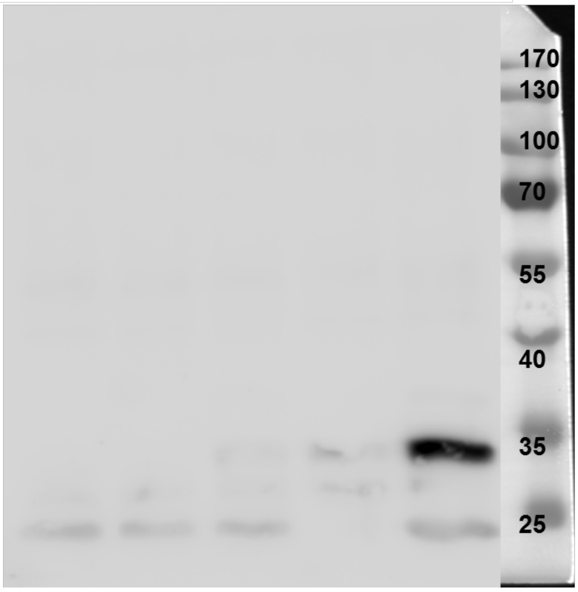

Fig. 5b α-DDK-IP; WB:α-DDK

CD248-DDK	-	+	-	+	+
Gal3	-	-	+	+	+
Anti-DDK Ab	+	+	+	-	+
IgG	-	-	-	+	-

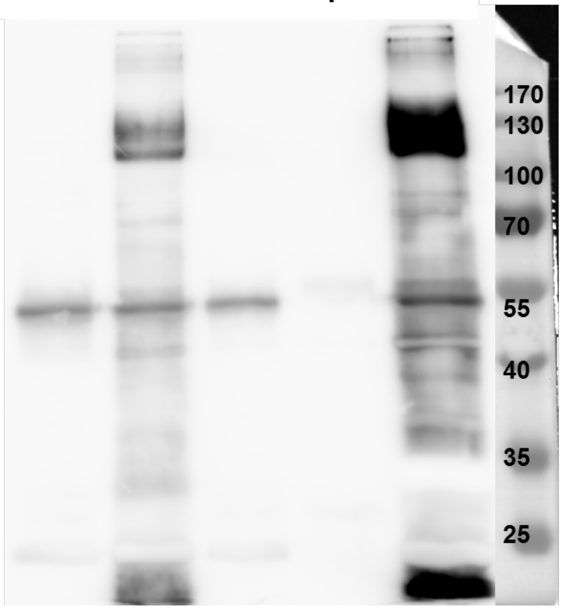

Fig. 5c

<u>α-Gal3-IP; WB:α-DDK</u>

CD248-DDK	+	+	-	+	-
Gal3	+	+	+	-	-
Anti-Gal3 Ab	+	-	+	+	+
IgG	+	+	-	-	-

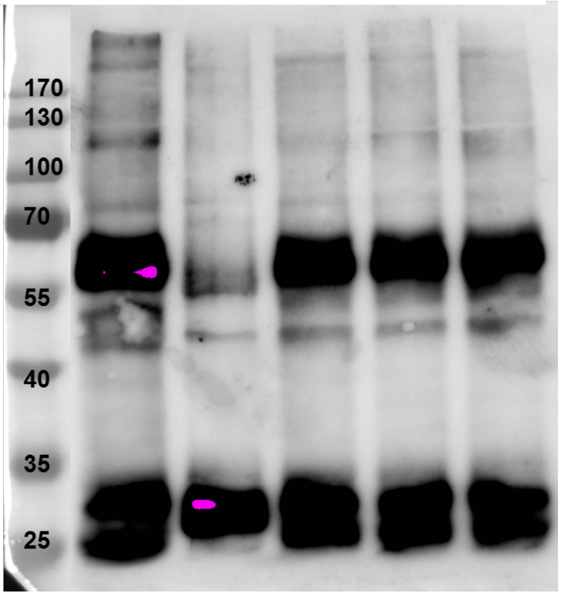

Fig 5c

<u>α-Gal3-IP; WB:α-Gal3</u>

C248-DDK	+	+	-	+	-
Gal3	+	+	+	-	-
Anti-Gal3 Ab	+	-	+	+	+
IgG	-	+	-	-	-

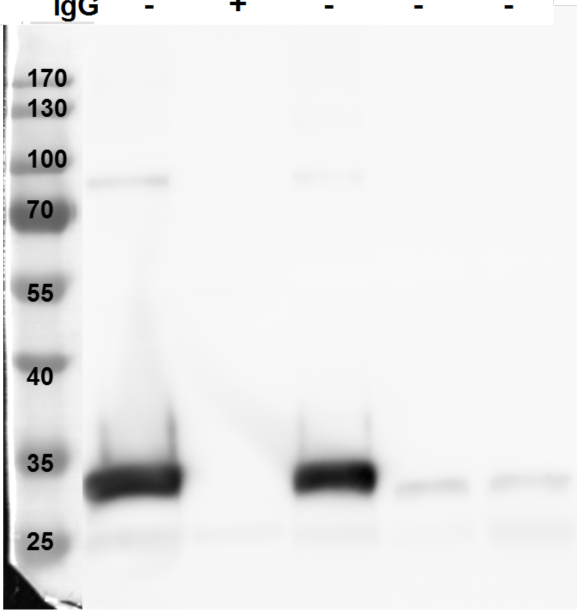

Fig. 5g PCR

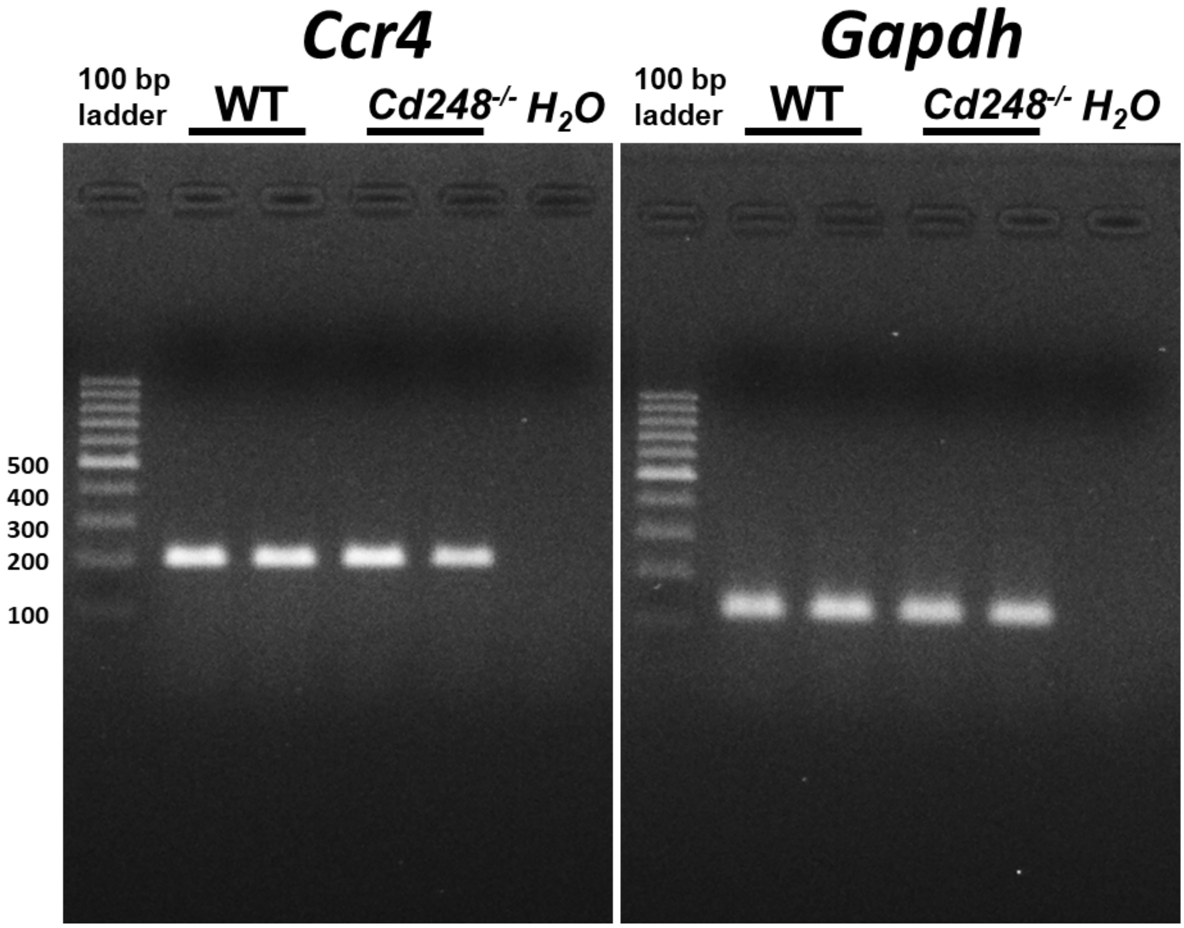
Ccr4
Gapdh
100 bp ladder
WT
Cd248-/-
H2O
100 bp ladder
WT
Cd248-/-
H2O
500
400
300
200
100
